# Circular‐Gate Nanoscale Air Channel Transistors: Achieving ultralow Subthreshold Swing and Working Voltage

**DOI:** 10.1002/advs.202410734

**Published:** 2024-12-25

**Authors:** Haiquan Zhao, Feiliang Chen, Yazhou Wei, Lixin Sun, Ruihan Huang, Xiangdong Wang, Fan Yang, Hao Jiang, Yang Liu, Mo Li, Jian Zhang

**Affiliations:** ^1^ School of Electronic Science and Engineering University of Electronic Science and Technology of China Chengdu 611731 China

**Keywords:** high on/off ratio, high temperature and irradiation resistance, nanoscale air channel transistors, ultralow subthreshold swing, ultralow working voltage

## Abstract

As electronics advance toward higher performance and adaptability in extreme environments, traditional metal‐oxide‐semiconductor field‐effect transistors (MOSFETs) face challenges due to physical constraints such as Boltzmann's law and short‐channel effects. Nanoscale air channel transistors (NACTs) present a promising alternative, leveraging their vacuum‐like channel and Fowler–Nordheim tunneling characteristics. In this study, a novel circular gate NACT (CG‐NACT) is purposed, fabricated on a 4‐inch silicon‐based wafer using a CMOS‐compatible process. By employing an innovative gate control mechanism, the transistors achieve an ultralow SS of only 0.15 mV dec^−1^ and maintain the average SS remained at 1.5 mV dec^−1^ over three decades of drain current. Additionally, our CG‐NACTs deliver milliamper‐level drain current at a low drain voltage of 0.7 V, with a maximum on/off ratio of 7.82×10^6^. Notably, CG‐NACTs remain highly stable even at high temperatures of up to 150 °C and under irradiation. Furthermore, the practical application of CG‐NACTs is successfully implemented by designing an inverter circuit for the first time.

## Graphical abstract

Our CMOS‐compatible nanoscale air channel transistor (NACT), fabricated on 4‐inch silicon wafers, achieves 0.15 mV dec^−1^ ultralow subthreshold swing via innovative gate control. Operating at 0.7V, it exhibits outstanding milliampere‐level drain current and a maximum on/off ratio of 7.82×10^6^, surpassing traditional NACT limits. It maintains high stability under harsh conditions. An inverter circuit is demonstrated for the first time.

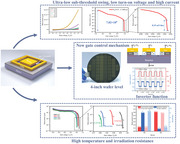

## 1 Introduction

The proliferation of emerging markets, such as the Internet of Things,^[^ ^1^, ^2^, ^3^, ^4^ ^]^ aerospace,^[^ ^5^, ^6^ ^]^ and 6G communication,^[^ ^7^, ^8^, ^9^, ^10^ ^]^ has sparked a surge in demand for devices with high energy efficiency and fast switching speed. One of the critical parameters determining the performance of these devices is the subthreshold swing (SS), which quantifies the gate voltage required to achieve a tenfold change in drain current, reflecting the device's switching transition characteristics and sensitivity to gate voltage.^[^ ^11^ ^]^ Nevertheless, the conventional metal‐oxide‐semiconductor field‐effect transistors (MOSFETs), which operate based on the thermionic emission‐based transport mechanism, face impediments in achieving an SS below 60 mV dec^−1^ at room temperature due to the limitations imposed by the Boltzmann's tyranny.^[^ ^12^ ^]^ Additionally, MOSFETs face limitations in operating at ultralow voltages (<0.7 V) due to their inherent physical constraints and conventional manufacturing techniques. Hence, it is imperative to explore new physical principles and device structures to achieve low SS values and operate at ultralow voltages. Advancing beyond the physical limits of conventional devices requires investigating new concepts, materials, and operational mechanisms. Potential candidates include negative capacitance field‐effect transistor,^[^ ^13^, ^14^, ^15^, ^16^, ^17^ ^]^ tunneling field‐effect transistors,^[^ ^16^, ^18^, ^19^, ^20^, ^21^, ^22^ ^]^ impact ionization field‐effect transistor,^[^ ^11^, ^23^, ^24^, ^25^ ^]^ 2D material‐based electronic devices^[^ ^26^, ^27^, ^28^ ^]^ and spintronics devices.^[^ ^29^, ^30^, ^31^ ^]^

As emerging devices, the nanoscale air‐channel transistors (NACTs) have attracted enormous attention. They utilize the nanoscale air channels as the carrier transport channel, which is smaller than the mean free path (MFP) of electrons in the air (≈68 nm), enabling scattering‐free transport of electrons in close‐to‐vacuum conditions.^[^ ^32^, ^33^, ^34^ ^]^ Leveraging their near‐ballistic transport characteristics, NACTs exhibit rapid response, high‐frequency capability, as well as resilience to elevated temperatures and radiation compared to conventional solid‐state devices.^[^ ^35^, ^36^ ^]^ The inherent insulation provided by the air channel results in outstanding on/off current ratios for NACTs. Moreover, based on the Fowler–Nordheim (FN) tunneling mechanism, the NACTs have promising potential for achieving low SS. However, despite the theoretical advantages of NACTs, their electrical performance currently falls short of expectations. **Table** **1** presents the performance of typical reported NACTs,^[^ ^37^, ^38^, ^39^, ^40^, ^41^, ^42^ ^]^ revealing a minimum SS of 50 mV dec^−1^, with an air channel of only 7 nm realized by a special nanosphere lithography technique.^[^ ^39^ ^]^ For the other proposed NACTs, their SS values far exceed 60 mV dec^−1^ failing to fully exploit the theoretical potential. Furthermore, the relatively low drain currents (microampere‐level) and higher operating voltages (>1 V) of current NACTs severely limit their practical use. These limitations are primarily due to the gate control mechanism, which is difficult to effectively influence the FN tunneling and electron trajectory, resulting in generally higher SS. To fully unlock the potential of NACTs, further research and development efforts are imperative.

**Table 1:** Electrical performances of typical reported NACTs.

Device Structure	Cathode Material	Fabrication Method	Channel Length [nm]	I_DS_ @V_DS_ [µA @ V]	On/Off	SS [mV dec^−1^]
Planar NACT^[^ ^37^ ^]^	n‐Si	Photoresist ashing	150	10@8.9	e6	4200
Planar NACT^[^ ^38^ ^]^	Si nanowire	Photoresist ashing	sub‐50	3@5	≈e4	>500
Planar NACT^[^ ^39^ ^]^	Metal	Nanosphere	6.88	<1@1.2	<e6	<50
Planar NACT^[^ ^40^ ^]^	Au	E‐Beam lithography	sub‐100	10@10	e4	720
Vertical NACT^[^ ^41^ ^]^	SiC	Reactive ion etching	200	11.4@20	126	>5000
Vertical NACT^[^ ^42^ ^]^	p‐Si	Focused ion beam	70	0.1@2	500	>400
This work (D1)	n‐Si	DRIE+BOE	50	7990 @0.7	7.8e6	0.15

In this study, we proposed a novel silicon‐based circular gate NACTs (CG‐NACTs). Their working principle was elucidated through simulations and experimental investigations. The innovative aspect of our NACT design lies in its unique gate control mechanism, whereby the gate modulates the electron concentration within the source region (the gate can extract or provide electrons from the source), thus effectively governing the current level between the source and drain. These CG‐NACTs were successfully fabricated on a 4‐inch silicon wafer with CMOS‐compatible technology, featuring an air channel width of 50 nm. They exhibited exceptional performance characteristics, showcasing an ultralow SS of 0.15 mV dec^−1^, making it one of the reported transistors with the lowest SS. Additionally, an SS variation of merely 1.5 mV dec^−1^ over three orders of magnitude and an impressive maximum switching ratio of 7.82×10^6^ were obtained. Our CG‐NACT operated efficiently at a low drain voltage of 0.7 V, delivering high current output. They also exhibited stable operation even under high‐temperature conditions reaching up to 150 °C, their SS and other performances remained slightly unaffected by the elevated temperatures. Long‐term testing further validated the excellent stability of the CG‐NACTs. Furthermore, we successfully demonstrated, for the first time, the practical functionality of an inverter based on the proposed transistors experimentally. These significant advances highlight the potential of NACTs to push the boundaries of conventional solid‐state devices, injecting a lasting impetus to the future development of electronics.

## 2 Results

### 2.1 Device Structure and Fabrication

**Figure 1:**
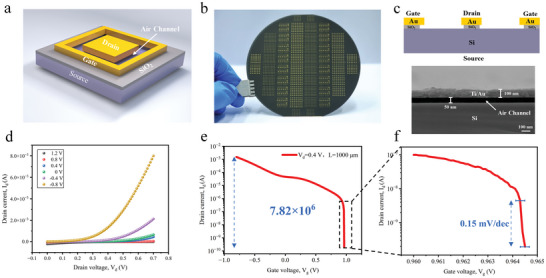
Silicon‐based CG‐NACTs. a) Schematic diagram of the CG‐NACT. b) Photograph of the CG‐NACT fabricated on a 4‐inch Si wafer. c) Cross‐section and SEM image of the CG‐NACT after BOE etching. Scale bar, 200 nm. d) Output characteristic of the D1 (BOE 10s, effective emitting areas: 600 µm^2^) device. e,f) Transfer characteristic of D1 (BOE 10s, effective emitting areas: 600 µm^2^) device (e) and its magnified Figure (f).

The device structure of the CG‐NACTs is depicted in **Figure** **1**a. The source was fabricated from highly doped *n*‐type Si, while the drain electrode and gate electrode were made of gold. The channel between the gate and drain measured 3 µm, and the air channel of 50 nm between the gate and source (Figure 1a). The detailed fabrication process, as detailed in Figure S1 (Supporting Information). The gate and drain were aligned in the same plane (Figure S2, Supporting Information). The CG‐NACTs fabricated on a 4‐inch silicon wafer as shown in Figure 1b. Buffered oxide etchant (BOE) was utilized to remove the silicon dioxide and enabled effective management of the emission area of the NACTs, thereby influencing their electrical performance. The post‐BOE‐etching structure of the CG‐NACT and the corresponding Scanning Electron Microscope (SEM) image are presented in Figure 1c.

In the design of our CG‐NACTs, with the gate and drain electrodes aligned on a common plane and perpendicular to the source, the air channel size was only determined by the thickness of the oxide layer, as illustrated in Figure 1a. Precise control of the air channel can be achieved by adjusting the thickness of the silicon dioxide layer, presenting a cost‐effective technique for large‐scale production. Additionally, the sandwich‐like configuration facilitated the utilization of various electrode materials for CG‐NACTs, through a simple coating deposition process, thereby enabling high drain current at low working voltages.

### 2.2 Electrical Performances and Electron Emission Mechanism

All experiments in this study were carried out under atmospheric conditions at room temperature unless specified otherwise. On the basis mentioned above, we fabricated the D1 device with a drain length of 1000 µm. After a 10 s wet etching process using Buffered Oxide Etch (BOE), the effective emitting area was measured to be 600 µm^2^. For detailed information regarding the etching rate, effective emitting area, and comparative data before and after etching, please refer to Figure S3 (Supporting Information). It is worth noting that the relatively large electrode area design in our devices was primarily intended to facilitate probe testing as well as the ease of demonstrating and verifying the functionality of this novel device and gate control mechanism. The larger area also aids in observing the wet etching rate and calculating the effective emitting area through scanning electron microscopy (SEM). Despite the initial larger area, the subsequent analysis indicates that scaling the device down to 36 µm^2^ does not significantly affect its electrical performance.

Figure 1d illustrates the output characteristics of the D1 device. Operating at a drain voltage of 0.7 V and a gate voltage of −0.8 V, D1 showcased a maximum drain current of 7.9 mA, marking it as the NACT with the highest reported drain current to date (as summarized in Table 1). It is evident from Table 1 that the majority of other NACTs operate within voltage ranges of 8–20 V and struggle to function below 1 V. In contrast, our device can achieve high currents at 0.7 V, a distinctive advantage that enhances its suitability for integration with other microdevices on the same low‐voltage platform, significantly broadening its application scenarios. Additionally, a decrease in gate voltage corresponded to a gradual increase in the current, indicating the impressive negative gate control capability of the proposed CG‐NACT, as Figure 1d illustrates.

As presented in Figure 1e, at a drain voltage of 0.4 V, the D1 device demonstrated a maximum on/off ratio of 7.82×10^6^ and an impressively low SS of only 0.15 mV dec^−1^. A comparison in Figure S4 (Supporting Information) clearly indicated that its SS is significantly lower than that of other reported low SS transistors, including direct TFETs, NC‐FETs, and I^2^FET.^[^ ^13^, ^14^, ^15^, ^16^ ^]^ The on/off ratio of the CG‐NACT was only lower than that of TFET, further emphasizing its potential for widespread applications.

**Figure 2:**
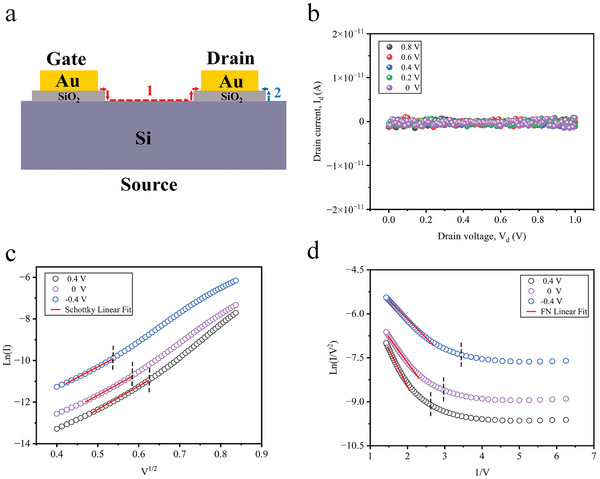
Validation of electron transport paths and emission mechanisms of the CG‐NACT. a,b) Schematic diagram (a) and drain current at different gate voltages (b) of the specially treated transistor. Electron transport paths, labeled 1 and 2, represent potential routes through which electrons can possibly propagate in the transistor. c,d) Schottky linear fit (c) and FN linear fit (d) of the output characteristic of the CG‐NACT (D1) with gate voltages of 0.4, 0, and −0 .4V.

To validate that the observed emission phenomena originated from electron transmission through the nanoscale air channel rather than surface conduction on the oxide layer, a device etching strategy was employed. By adjusting the parameters of deep reactive ion etching (DRIE), the oxide layer beneath the gate and drain regions was intentionally extended beyond their dimensions by several tens of nanometers. This DRIE etching process effectively prevented electron transmission through the air channel, thus obstructing their flow toward the drain of the NACT. The structural diagram and SEM images of the specially treated transistor were presented in **Figures** **2**a and S5 (Supporting Information), respectively as depicted in Figure 2b, the drain current remained remarkably lower than 1×10^−11^ A, indicating a negligible current flow. This outcome confirmed that the measured current of the D1 device did not arise from electron conduction on the oxide surface, but was indeed attributable to electron transmission through the air channel.

Based on this foundation, the electron emission mechanism of the CG‐NACT is explained. From Figure 2c,d, it can be observed that at a gate voltage of 0.4 V, the electron emission between the source and drain followed the Schottky emission mechanism when the drain voltage was below 0.37 V. However, FN tunneling gradually became the dominant mechanism for electron emission with higher drain voltages. The equations describing the Schottky emission and FN tunneling emission can be found in Supporting Information. Furthermore, as the gate voltage gradually decreased from 0.4 to −0.4 V, the drain voltage required for FN tunneling also reduced from 0.37 to 0.29 V.

### 2.3 Novel Gate Control Mechanism

Herein, we elucidate the gate control mechanism of the CG‐NACT to help understand the underlying principles for its superior performance. Currently, NACTs can be categorized into two main types: planar‐structured NACTs with back gates^[^ ^37^ ^]^ and vertical‐structured NACTs with intermediate gates.^[^ ^42^ ^]^ The gate was employed to modulate the electric field distribution within the air channel, thereby influencing the FN tunneling probability and electron transport.^[^ ^40^, ^41^, ^42^ ^]^ However, they typically operated at relatively high drain voltages (as shown in Table 1), leading to a strong electric field within the nanoscale air channel. As a result, a small adjustment in the gate voltage was insufficient to induce a significant change in the electric field of the air channel, resulting in higher SS.

In order to enhance the gate control ability and reduce the SS, the current predominant approach in NACTs involves reducing the drain voltage to enhance the impact of gate voltage variations on the electric field within the air channel.^[^ ^38^, ^39^ ^]^ Based on the FN tunneling mechanism, the reduction of the air channel length between the source and drain can significantly reduce the required drain voltage. As shown in Table I, both planar and vertical structured NACTs demonstrated a decrease in the drain voltage as air channel length gradually diminished, thereby augmenting the influence of the gate on the electric field and consequently reducing the SS. However, even with the reduction of the air channel length to 7 nm through specialized processing, the SS of NACT remained at ≈50 mV dec^−1^, indicating limited improvement.^[^ ^39^ ^]^

**Figure 3:**
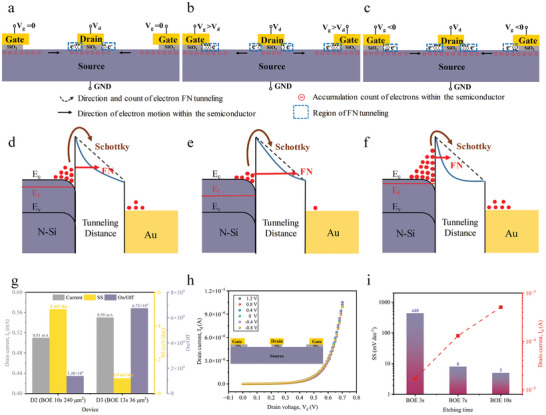
The gate control mechanism of CG‐NACT. a–c) Schematic diagram illustrating the gate control mechanism when V_g_ = 0 (a), 0< V_d_ <V_g_ (b) and V_g_< 0<V_d_ (c). d–f) Energy band diagrams of the drain and source of the transistor when V_g_ = 0 (d), 0< V_d_ <V_g_ (e), and V_g_< 0<V_d_ (f). E_c_, E_f_, and E_v_ denote the conduction band, Fermi level, and valence band, respectively. g) Comparative plots of drain current, SS, and On/Off ratio for D2 (BOE 10s, effective emitting areas: 240 µm^2^) and D3 (BOE 13s, effective emitting areas: 36 µm^2^) devices with different effective emitting areas. h) Output characteristic of transistors fabricated by the photoresist overlay process and BOE etching. Where the inset is the schematic of its structure. i) Minimum SS (black, left axis) and drain current (red, right axis) of the transistor (D2 device) for different etching times.

In this paper, we successfully demonstrate an ultralow SS in our CG‐NACT with an air channel of 50 nm. The primary innovation of the CG‐NACT lies in the novel gate control mechanism we designed. Due to the optimization of the device structure, the gate modulated the electron concentration at the source region (the gate extracts or provides electrons from the source), rather than modulating the electric field and electron trajectories in the air channel, as illustrated in **Figure** **3**a–c. Specifically, in the absence of gate voltage, the drain current in CG‐NACTs primarily originated from FN tunneling between the source and drain (FN_ds_), as Figure 3a depicted. However, when a positive gate voltage exceeded the drain voltage, FN tunneling between the gate and source (FN_gs_) became more favorable. Electrons tend to move toward regions offering easier tunneling pathways,^[^ ^43^ ^]^ leading them to migrate from the source region beneath the drain to the area below the gate rather than tunneling upward, as illustrated in Figure 3b. This migration resulted in a significant decrease in the electron concentration of the source region beneath the drain, thus suppressing the FN_ds_. Conversely, the application of a negative gate voltage prompted a significant emission of electrons from the gate toward the lower source, facilitating their diffusion into the drain region and thereby enhancing the FN_ds_, as shown in Figure 3c. Notably, the FN tunneling mechanism in semiconductors exhibits an exponential relationship between voltage changes and electron concentration. This electron concentration, in turn, exerts an exponential influence on the drain current. Thus, even slight fluctuations in gate voltage can lead to a substantial alteration in the electron concentration at the source, consequently leading to a significant variation in the drain current. This characteristic enabled the CG‐NACT to achieve an extremely low SS.

The validation of the proposed gate control mechanism was further reinforced through simulations using Silvaco software to investigate the electron concentration in NACT under different gate voltages, as depicted in Figures S6,S7 (Supporting Information). The simulation results align well with the theoretical analysis. It's noteworthy that the Silvaco software did not provide electron trajectory visualization for tunneling events, the simulated electron concentration represented an instantaneous snapshot of the process. Additionally, the transfer characteristics of the CG‐NACTs were also simulated, as presented in Figure S8 (Supporting Information). The simulation results also showed good consistency with the experimental results, demonstrating that the drain current decreased sharply with a positive gate voltage and enhanced with a negative gate voltage.

From an energy level perspective, changes in electron concentration could affect the shape and width of the air channel barrier. As depicted in Figure 3d, when maintaining a constant drain voltage and no gate voltage is applied, electrons tunnel from the source to the drain. However, when the applied gate voltage surpasses the drain voltage, a majority of electrons are diverted to the gate instead of tunneling to the drain. This leads to a reduction in electron accumulation, a weakening of the image force, and an expansion of the barrier width, leading to a decrease in FN tunneling between source and drain, as illustrated in Figure 3e. Conversely, when a negative gate voltage is applied, a significant emission of electrons from the gate toward the source occurs. These electrons rapidly migrated to the source region, causing a narrowing of the barrier and enhancing the probability of tunneling to the drain, as shown in Figure 3f.

In CG‐NACTs, the electron extraction or provision capability of the gate electrode is crucial. This ability primarily arises from the device's structural design that results in air channels rather than relying solely on the emitting area. Theoretically, based on the field emission formula, the field emission current exhibits an exponential relationship with the channel length, while showing only a linear correlation with the emitting area. Thus, the channel size's impact on the device's performance is significantly greater than that of the emitting area. This conclusion aligns with the findings reported in previous studies.^[^ ^43^, ^44^ ^]^ To validate that the emitting area is not the primary factor driving the superior electrical performance of our NACTs, we fabricated devices with two sizes: one with a 400 µm edge length resulting in an effective emitting area of 240 µm^2^ after 10s of BOE etching (D2), and another with a 50 µm edge length resulting in an effective emitting area of 36 µm^2^ after 13s of BOE etching (D3). The transfer and output characteristic curves of D2 and D3 are illustrated in Figures S9,S10 (Supporting Information). Although D3 has only 15% of the area of D2, it outperforms D2 electrically in terms of output current, SS, and on/off ratio (see Figure 3g).

Furthermore, the warping of device electrode edges due to photolithographic stripping leads to the formation of an initial air channel ≈90 nm during etching, which then erodes inward by ≈150 nm before stabilizing at the designed 50 nm (Figure S13, Supporting Information). Simulations reveal that a deviation of 40 nm in the channel size can cause a variation in current by at least 6×10^5^ times (see Figure S11, Supporting Information). Previous literature has already addressed the conclusions regarding the impact of the channel on field emission.^[^ ^33^ ^]^ When the air channel exceeds the intended 50 nm, the gate's ability to effectively control electron extraction or delivery is compromised, resulting in weak gate control.

We experimentally verified the impact of air channels on the electrical performance of the devices. Experimentally, we re‐fabricated the D2 device using a photoresist overlay process to selectively etch the SiO_2_ beneath the drain while preserving it beneath the gate. As shown in Figure 3h, this process effectively prevented FN tunneling between the gate and the source, rendering the device without gate control capability. The lack of gate control of the CG‐NACTs in this case was confirmed by Silvaco simulations, closely matching the experimental results (refer to Figure S12, Supporting Information). Subsequently, we varied the duration of BOE etching to obtain different etching depths, corresponding to achieving different air channel lengths, as shown in Figure S13 (Supporting Information). With a 3s etching time, the air channel length (d) measures ≈70 nm (Figure S13, Supporting Information), resulting in weaker gate control with a maximum SS of 440 mV dec^−1^ and a current of only 0.017 mA at a 0.7 V drain voltage (Figure 3i and Figure S14, Supporting Information). When the etching time was extended to 7s, the air channel length decreased to ≈55 nm, significantly improving gate control: the SS was optimized to 8 mV dec^−1^ (a 55‐fold improvement) and the current increased to 0.14 mA (an 8.2‐fold increase, as shown in Figure S15, Supporting Information). Further extending the etching time to 10 s, the air channel length decreases to 50 nm, but the SS improvement was only 1.6‐fold (5 mV dec^−1^), and the current increased by ≈3.6‐fold (0.51 mA), as shown in Figure S8 (Supporting Information). In summary, while the emitting area does influence device performance, channel length is the key factor affecting the gate control capability of the CG‐NACT.

The CG‐NACTs leverage their advanced gate control mechanism to achieve both minimized SS and effective operation at ultralow voltages, yielding significant drain currents. This is because the gate can provide or extract numerous electrons to the source, greatly influencing the electron tunneling probability and consequently enhancing the drain current. The use of electrodes with low work functions and a reduction in air channel dimensions further contribute to improved drain currents and lower operational voltages, as described by the FN tunneling equation. Additionally, when electrodes are formed through electron‐beam vaporization of metal, some degree of uniform roughness is inevitable, as illustrated in Figure S16 (Supporting Information). For NACTs with air channels on the order of tens of nanometers, this nanoscale roughness can act as a localized field enhancement factor. This “roughness effect” helps reduce the operational voltage required for field emission. This unique “roughness effect” presents a novel avenue for further optimizing the devices.

While our devices may exhibit some gate leakage current (Figure S17, Supporting Information), a common challenge in vacuum devices, their substantial potential should not be overlooked. The practical value of our devices extends beyond power consumption to include crucial performances such as high‐speed operation, high‐frequency capabilities, high‐temperature resilience, and radiation resistance. For instance, Aerospace applications have even more stringent demands, requiring devices to maintain long‐term reliability under extreme temperatures and intense radiation, while supporting high‐current drive and high integration.^[^ ^41^ ^]^ Additionally, in advanced scientific fields like biochemical sensing, devices must achieve ultralow SS for high sensitivity, along with exceptional low‐voltage, high‐current characteristics.^[^ ^45^, ^46^, ^47^ ^]^ Traditional MOSFET devices struggle to meet these criteria, yet our new devices are expected to fulfill these critical benchmarks.

### 2.4 Long‐Term, High‐Temperature and Radiation Tolerance Stability

**Figure 4:**
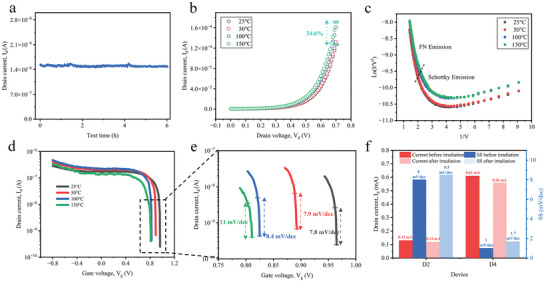
Long‐term and high‐temperature stability tests of the CG‐NACT (D2 device). a) Long‐term (6 h) stability tests at a gate voltage of 0.8 V and a drain voltage of 0.4 V. b) The output characteristics under the temperature from 25 to 150 °C. c) Corresponding FN plots under varying temperatures. d,e) Transfer characteristic of the CG‐NACT (D2) (d) and its local magnification (e) from 25 to 150 °C. f) Drain current and SS of D2 (BOE 7s, effective emitting areas: 168 µm^2^) and D4 (BOE 10s, effective emitting areas: 360 µm^2^) devices before and after neutron irradiation with a total dose of 1×10^10^ n cm^−2^.

To evaluate the long‐term and high‐temperature stability of the proposed CG‐NACTs, we conducted experiments with the D2 device (with a BOE etching time of 7 s). **Figure** **4**a demonstrates its endurance when operated at constant voltages for 6 h in the room temperature atmospheric environment. The gate voltage was maintained at 0.8 V and the drain voltage was set at 0.4 V. Throughout the testing period, the current‐time curve of the D2 device exhibited a very modest increase from 1.48 to 1.53 µA, representing a mere 3.38% increment, which demonstrated remarkable stability. Several factors contributed to the superior stability. Firstly, our CG‐NACTs operated at reduced working voltages, effectively mitigating the damage from electron collisions to the oxide layer and electrodes.^[^ ^44^ ^]^ Moreover, the construction of the source and drain in a plane‐to‐plane configuration ensured a more uniform electric field distribution, thereby reducing the risk of device failure and promoting stable operation.

In Figure 4b, the *I–V* characteristics of the D2 (BOE 7s, effective emitting areas: 168 µm^2^) device under a gate voltage of ‐0.8 V and a drain voltage ranging from 0 to 0.7 V were obtained across a temperature range of 25 to 150 °C. As the temperature increased, the drain current of the device gradually rose from 0.127 mA at 25 °C to 0.171 mA at 150 °C, indicating an increase of ≈34.6%. Despite this increase, the D2 (BOE 7s, effective emitting areas: 168 µm^2^) device still operated properly, unlike CMOS devices that typically fail ≈150 °C. The observed increase in the drain current can be attributed to two main factors. Firstly, water vapor adsorption on the electrode surfaces may have played a significant role.^[^ ^35^ ^]^ As depicted in Figure 4b,c, the current change was minimal when the temperature was below 100 °C. However, a substantial change occurred when the temperature reached 100 °C, leading to an increased probability of FN tunneling and subsequently boosting the drain current.^[^ ^33^ ^]^ To further address the impact of moisture on the electrical performance of the device, packaging treatment could be considered. Second, while FN tunneling was not temperature‐sensitive, the device also exhibited Schottky emission, which was positively correlated with temperature. The enhanced Schottky emission effect at higher temperatures provided more free electrons, contributing to the overall increase in the drain current.

The transfer characteristics shown in Figures 4d,e illustrated that the minimum SS of the D2 (BOE 7s, effective emitting areas: 168 µm^2^) device gradually increased with temperature at a drain voltage of 0.15 V. The SS deteriorated from 7.8 mV dec^−1^ at 25 °C to 11 mV dec^−1^ at 150 °C, representing an approximate degradation of 41%. Additionally, with higher temperature, the corresponding gate voltage for the minimum SS decreased from 0.96 to 0 .81V. The primary factor contributing to the SS degradation was the change in the carrier mobility of the source. As the temperature went up, internal phonon scattering occurred within the silicon, leading to a reduction in mobility and consequently affecting the drift–diffusion mechanism within the semiconductor. Although the ability of the gate to extract or provide electrons from or to the source remained constant, the movement of electrons within the source was affected. This weakened the gate's control over the electron concentration in the source. Despite that, the overall impact of temperature on the CG‐NACT was minimal, and the device maintained effective gate control even at a temperature of 150 °C. This superior performance exceeded most of the reported transistors with low SS,^[^ ^13^, ^14^, ^15^, ^16^, ^17^ ^]^ making it a promising option for high‐temperature applications.

Furthermore, irradiation resistance is a critical metric for assessing the reliability of electronic devices, especially those intended for operation in harsh environments.^[^ ^48^ ^]^ To address this, we conducted specific irradiation experiments to comprehensively evaluate the radiation resistance performance of the designed CG‐NACT devices. We subjected the D2 (BOE 7s, effective emitting areas: 168 µm^2^) device, to a neutron irradiation dose of 1×10^10^ n cm^−2^. As depicted in Figure S18 (Supporting Information), there was no significant degradation observed in the output and transfer characteristics of the devices before and after irradiation. Specifically, post‐irradiation, the device current only slightly decreased by ≈7%, with a 6% increase in SS (Figure 4f), indicating good radiation stability. To further validate this performance, we fabricated D4 device with a side length of 600 µm and an effective emission area of 360 µm^2^ (BOE 10s). As illustrated in Figure S19 (Supporting Information), the electrical performance of the D4 (BOE 10s, effective emitting areas: 360 µm^2^) device remained highly consistent before and after irradiation (Figure 4f). This reinforces the exceptional radiation resilience of CG‐NACT. This pivotal characteristic not only establishes a robust foundation for dependable device operation in harsh environments but also significantly broadens its potential applications in aerospace, nuclear energy, and other specialized domains.

### 2.5 Demonstration of an Inverter

**Figure 5:**
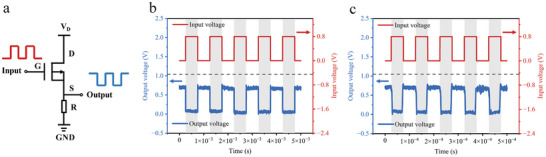
Demonstration of an inverter. a) Experimental scheme of the inverter based on the CG‐NACTs (D2 (BOE 7s, effective emitting areas: 168 µm^2^) device). The drain voltage was maintained constant while the gate voltage was a pulse square wave. b,c) Input voltage versus Output voltage when the drain was 0.8 V, the gate voltage was 0.8 V, and the frequency was 1 kHz (b) and 10 kHz (c).

In this study, we utilized the proposed CG‐NACT to construct a basic inverter circuit using the D2 (BOE 7s, effective emitting areas: 168 µm^2^) device, which serves as one of the fundamental units of circuits. To the best of our knowledge, this is the first experimental demonstration of the inverter circuit functionality using NACTs. As illustrated in **Figure** **5**a, the drain voltage was set at 0.8 V, and the gate input signal was a square wave with low and high levels of 0 and 0.8 V, respectively. The inverter circuit was constructed using D2 devices and resistors. The resistance in the inverter is externally connected. The input signal was supplied using an arbitrary signal generator, and the output signal was measured using an oscilloscope. Experimental results demonstrated that the inverter exhibited good inversion functionality under various input square wave conditions at 1 and 10 kHz, as shown in Figure 5b,c. The response time of the inverter was evaluated, revealing a response time of 3 µs when the rising and falling times of the 10 kHz input signal were both 3 µs, as shown in Figure S20 (Supporting Information). This indicates the CG‐NACT‐based inverter's capability to handle fast signals. It is worth noting that the 3 µs response time was limited by the parasitic parameters of the circuit, rather than the intrinsic switching speed of the device. To overcome this limitation, we utilized Silvaco software to numerically calculate the dynamic switching response of the device. The simulation results demonstrated that even with an input signal rise time reduced to 10 ns, the device continued to operate normally, as shown in Figure S21 (Supporting Information). Furthermore, by scaling down the device dimensions to minimize parasitic capacitance, the simulated switching response time of the device could be further improved. With an input signal rise time shortened to 100 ps, the device maintained normal operation, as illustrated in Figure S22 (Supporting Information). Future research aimed at further scaling down the device dimensions and optimizing the testing system should enhance the device's high‐speed performance.

## 3 Discussion

In summary, we introduced novel silicon‐based CG‐NACTs fabricated at the wafer scale using a CMOS‐compatible process. Leveraging a unique gate control mechanism which the gate modulated the electron concentration at the source region, the CG‐NACTs exhibited an exceptional ultralow SS of 0.15 mV dec^−1^, outperforming existing transistors. Moreover, these transistors realized milliamp‐level drain current and an on/off ratio greater than 10^6^ at a low working voltage of 0.7 V, effectively addressing longstanding challenges associated with NACTs. Even at 150 °C and under irradiation, the CG‐NACTs exhibited consistent operation with SS below 11 mV dec^−1^, highlighting their robustness and stability. Furthermore, the experimental functionality of an inverter based on the CG‐NACTs was achieved for the first time. These advances have not only solved the long‐standing bottleneck of NACT, but also paved the way for the future application of NACT in cutting‐edge fields.

## 4 Experimental Section

### Fabrication of the CG‐NACTs

The CG‐NACTs were fabricated using CMOS process‐compatible techniques, including thermal oxidation, photolithography, electron beam evaporation, deep reactive ion etching (DRIE), and buffered oxide etching (BOE). The detailed fabrication process can be found in Figure S1 (Supporting Information). First, a 50 nm thick silicon dioxide (SiO_2_) film was grown on a heavily doped *n*‐type silicon substrate through thermal oxidation. Photolithography was then employed to pattern the substrate, leaving a 3 µm channel between the gate and drain electrodes. To eliminate the remaining photoresist in the patterned region, a 30 s oxygen plasma treatment at 30 W power was performed. Next, a 10/90 nm thick titanium/gold (Ti/Au) electrode was deposited on the substrate using electron beam evaporation, of which the Ti layer was employed as the adhesion layer. The patterned region outside the photoresist and Ti/Au electrodes was then stripped using acetone and ethanol, resulting in a patterned Si/SiO_2_/Au multilayer structure. Subsequently, DRIE was performed using a mixture of CHF_3_ and O_2_ gases (CHF_3_:O_2_ = 2:1) at a pressure of 35 mtorr and an RIE power of 200 W. This step selectively removed the silicon dioxide in regions other than the gate‐drain. To form the air channel between the Au electrode and heavily doped *n*‐type silicon substrate, BOE was carried out using a solution ratio of NH_4_F:HF = 6:1. This process precisely eliminated the SiO_2_ below the drain and gate edges. It was worth noting that the area of the etched air channel could be accurately controlled by adjusting the duration of the BOE wet etching.

### Device Performance Measurement

Current–voltage (*I–V*) characteristics of CG‐NACTs were measured using the semiconductor parameters meter (FS‐Pro). The square wave voltage was generated by a signal generator (Tektronix AFG31152) and the output voltage was recorded by an oscilloscope (Tektronix MDO3104). All experiments were conducted under ambient conditions.

### Simulation

The TCAD software (Silvaco 2014) was used to simulate the impact of the FN tunneling mechanism on the electron concentration within the semiconductor source. By solving the Poisson equation, drift–diffusion equation, Shockley–Read–Hall recombination, FN tunneling formula, and other models, the transfer characteristic curve of the CG‐NACT was obtained. The material parameters such as the work function were set to the default values provided in Silvaco.

## Conflict of Interest

The authors declare no conflict of interest.

## Supporting information



Supporting Information

## Data Availability

The data that support the findings of this study are available from the corresponding author upon reasonable request.
