# Supplementary material for: Circular‐Gate Nanoscale Air Channel Transistors: Achieving ultralow Subthreshold Swing and Working Voltage
Source: Adv Sci (Weinh). 2024 Dec 25;12(7):2410734. doi: 10.1002/advs.202410734 (PMC11831514; doi:10.1002/advs.202410734)
Supplement: Supplementary file 1 — Supporting Information [file ADVS-12-2410734-s001.docx]

**Supporting Information**

**Circular-Gate Nanoscale Air Channel Transistors: Achieving Ultra-Low Subthreshold Swing and Working Voltage**

*Haiquan Zhao, Feiliang Chen, Yazhou Wei, Lixin Sun, Ruihan Huang, Xiangdong Wang, Fan Yang, Hao Jiang, Yang Liu, Mo Li* and Jian Zhang*

H. Q. Zhao, F. L. Chen, Y. Z. Wei, L. X. Sun, R. H. Huang, X. D. Wang, F. Yang, H. Jiang, Y. Liu, M. Li, J. Zhang

School of Electronic Science and Engineering, University of Electronic Science and Technology of China, Chengdu 611731, China.

E-mail: limo@uestc.edu.cn.

**Supporting Figures**


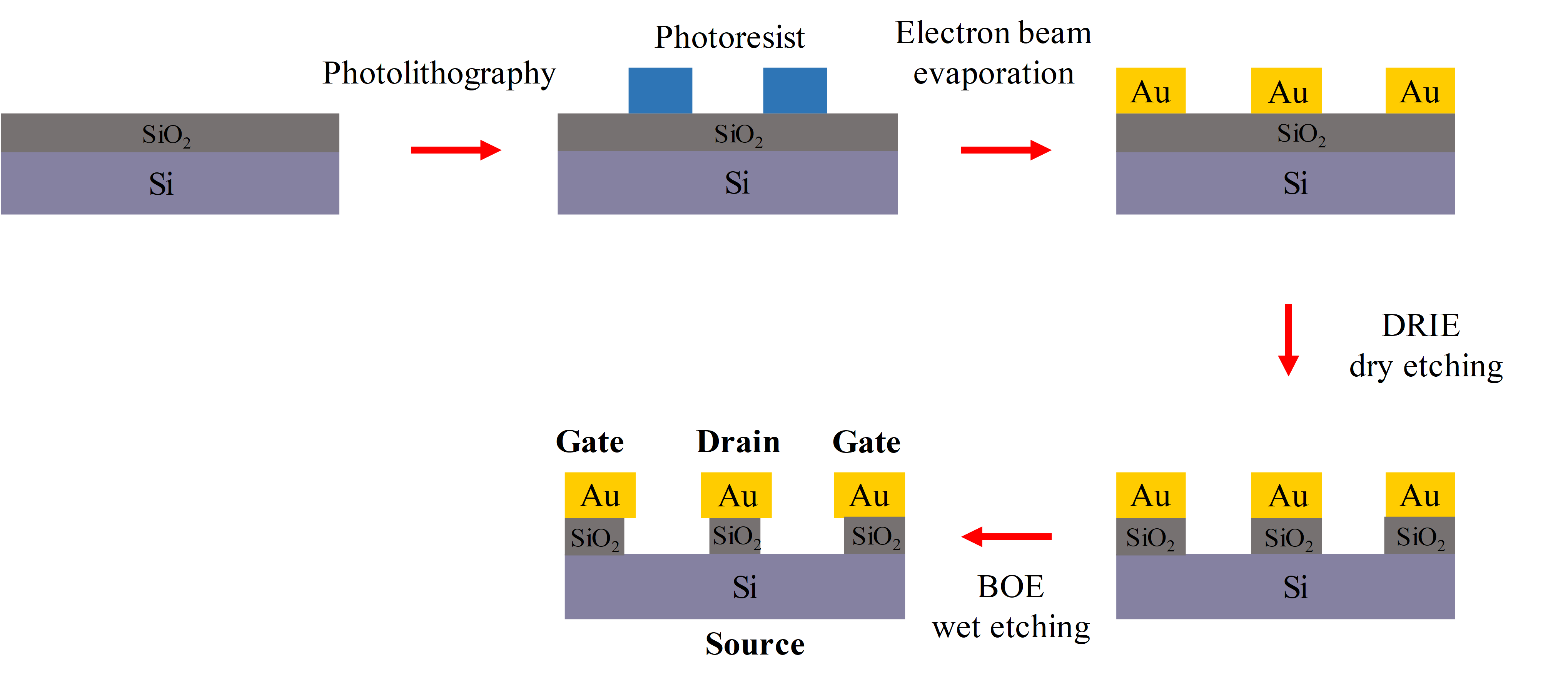


**Figure S1**. Fabrication process of the CG-NACTs.


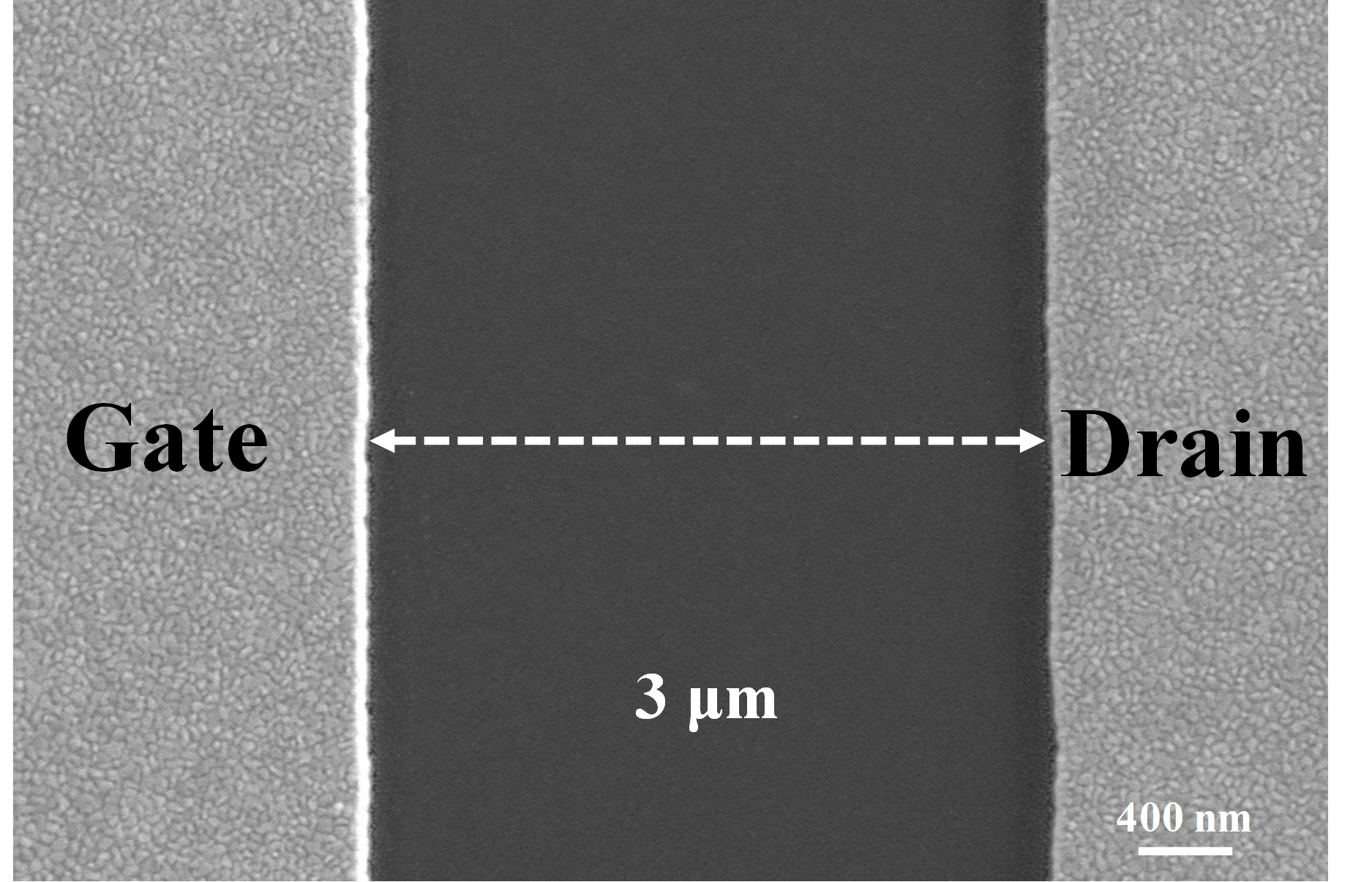


**Figure S2.** **SEM image of circular gate NACT with Ti/Au electrodes.**


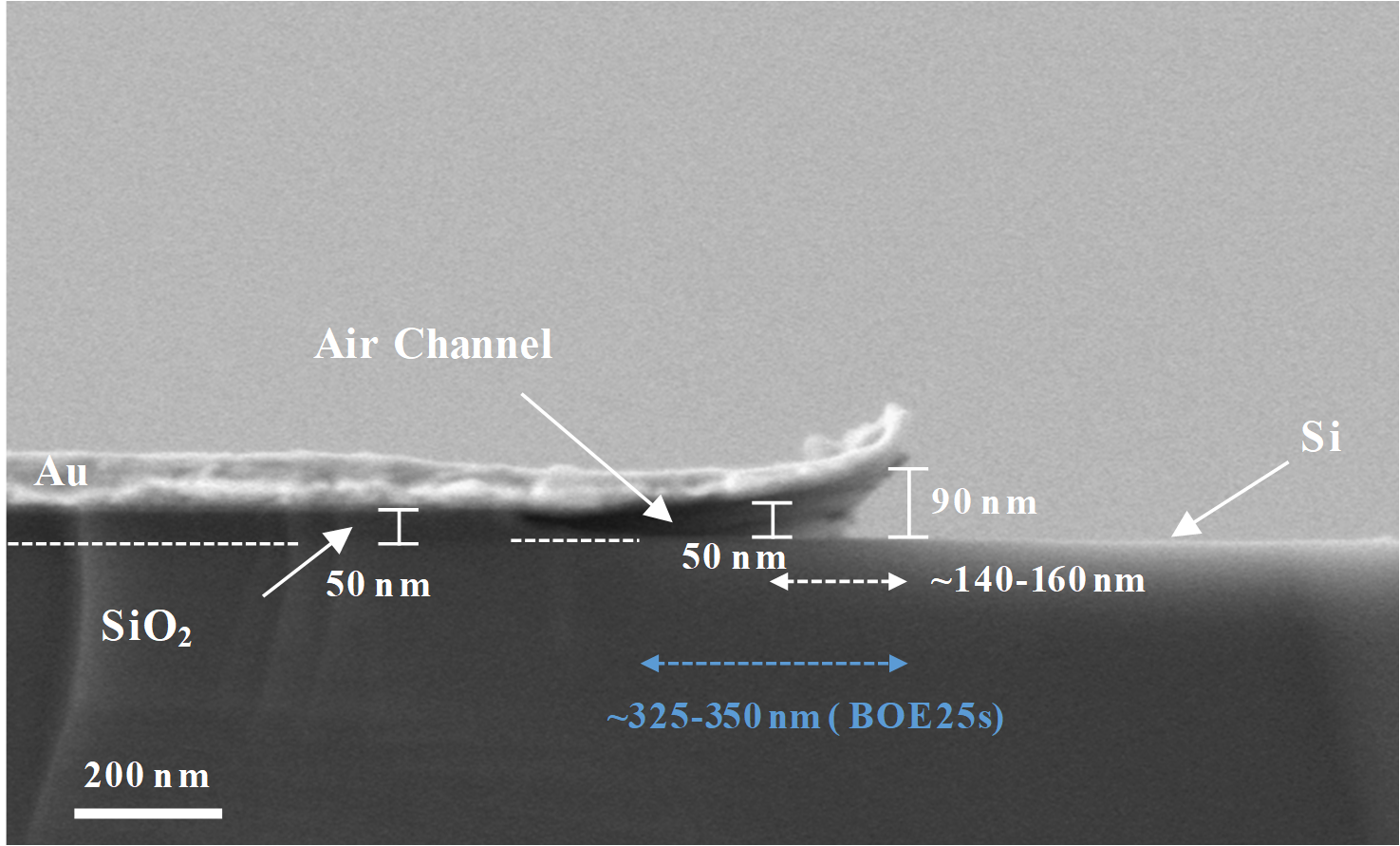


**Figure S3**. **SEM images of the CG-NACT after BOE wet etching (25s).** From the graph, it is evident that after wet etching with BOE for 25 seconds, an inward etch of approximately 350 nm occurred, with an etch rate ranging between 13-15 nm/s. The slight electrode elevation observed in the SEM image is attributed to the lithography stripping process during device fabrication. Additionally, due to the process, there is a slight uplift at the electrode edges, measuring approximately 90 nm in height, exceeding our intended design of 50 nm.


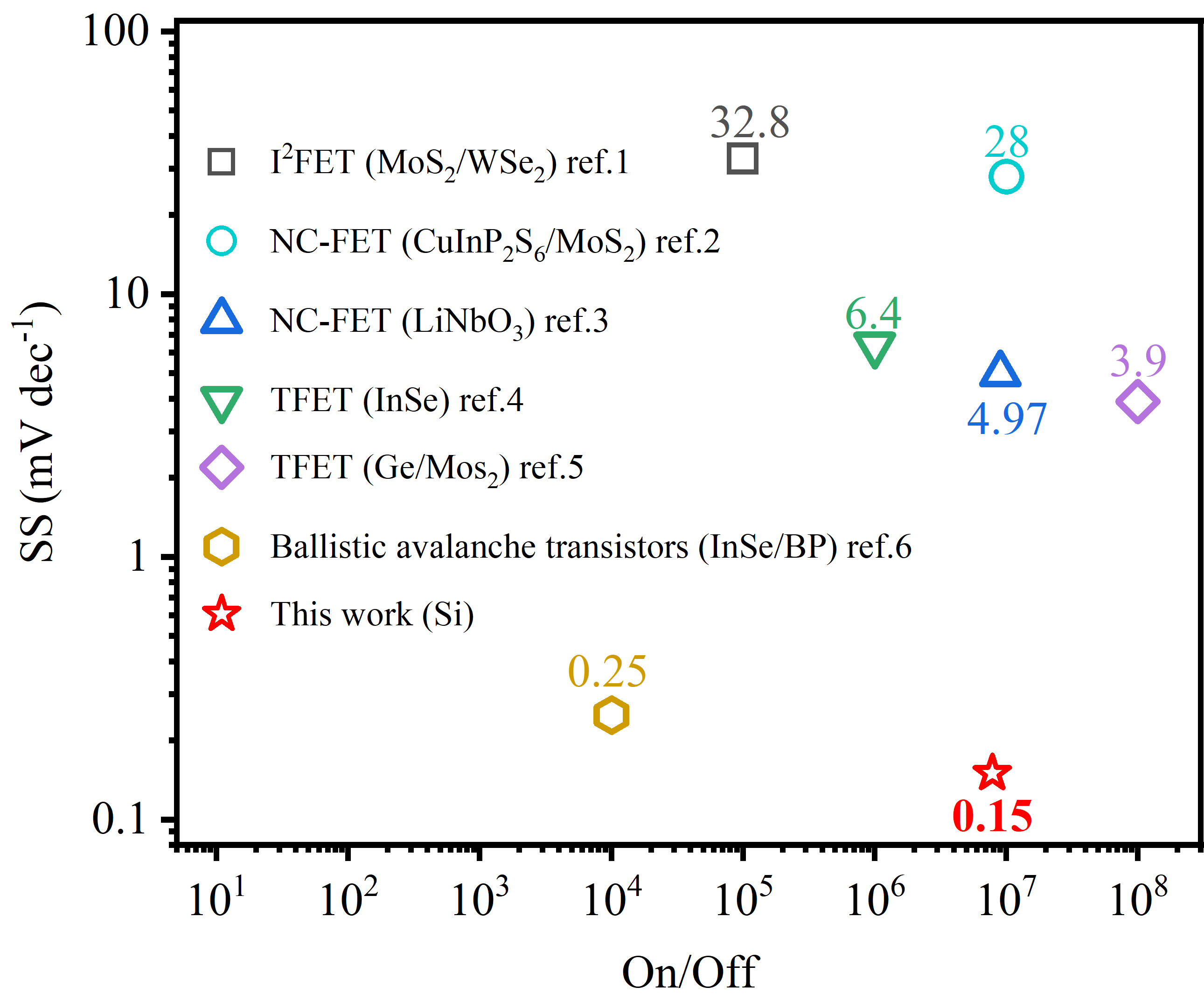


**Figure S4.** **Comparison of on/off ratios and minimum SS for different types of low SS transistors.** These include impact ionization field-effect transistor (I^2^FET), negative-capacitance field-effect transistor (NC-FET), tunnel field-effect transistor (TFET) and ballistic avalanche transistors.


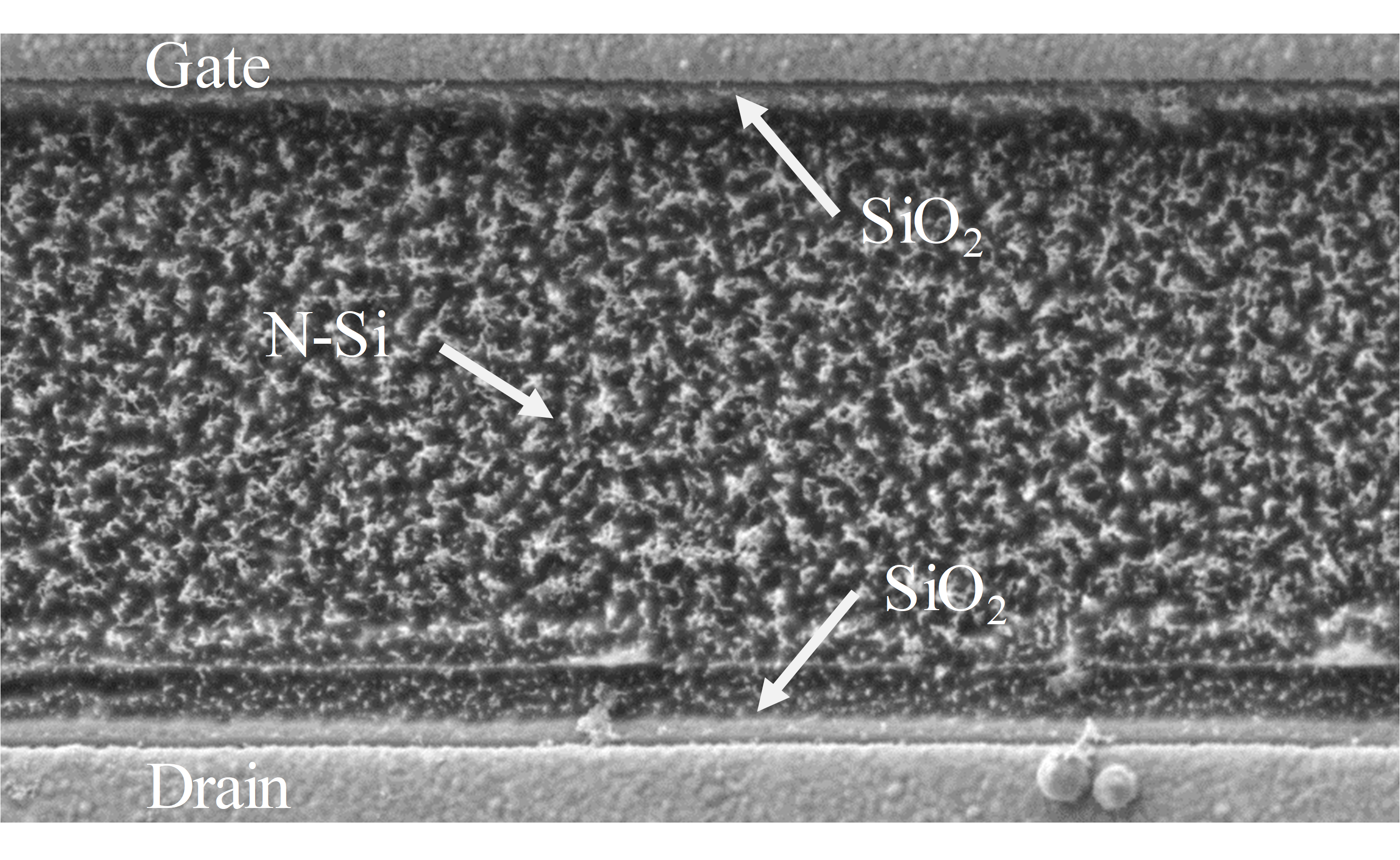


**Figure S5.** **SEM images of CG-NACT with silicon dioxide below both gate and drain.**


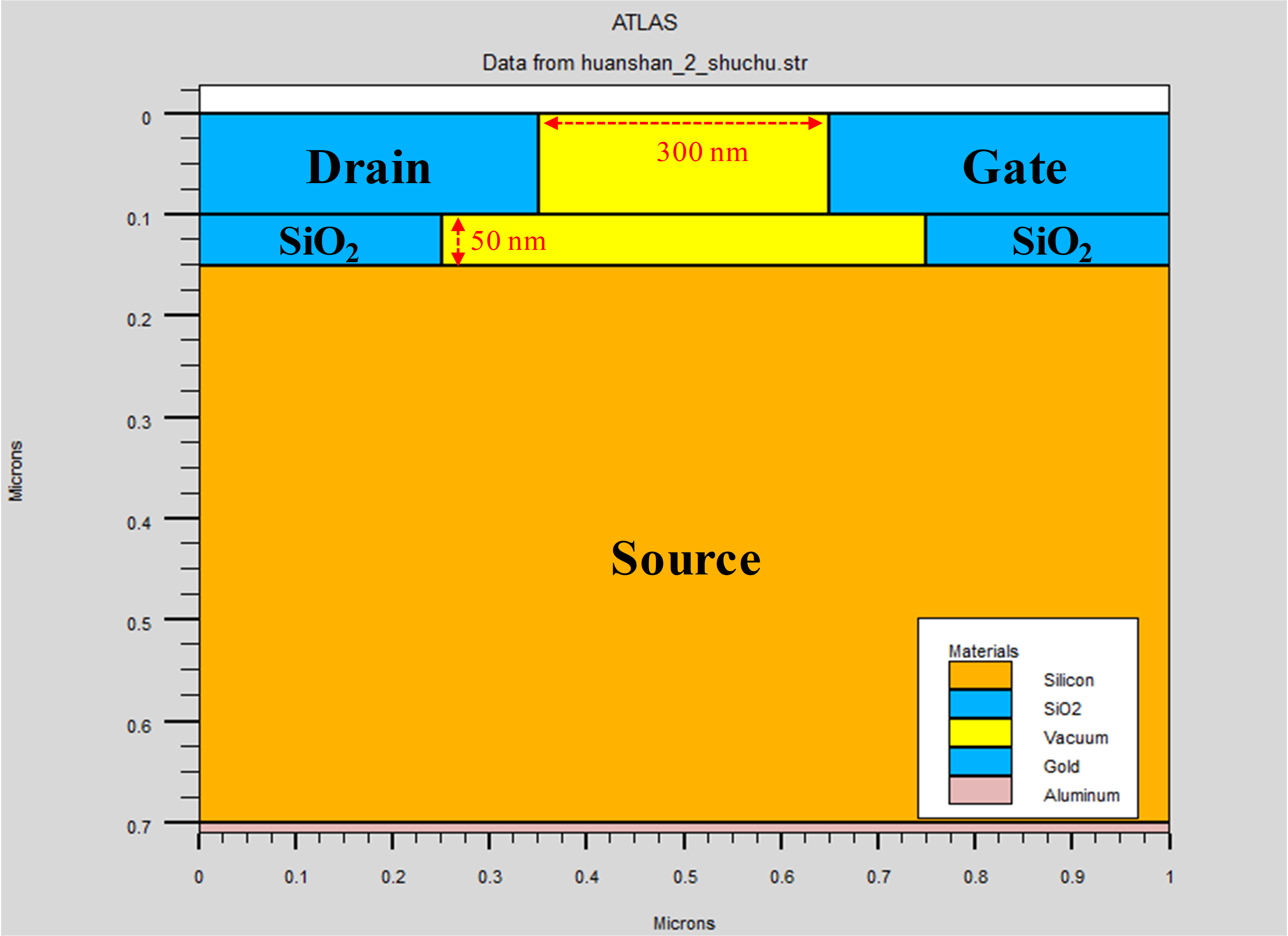


**Figure S6. Simulation structure of CG-NACT.** Considering its symmetrical structure, only half of the region is simulated. Furthermore, for better visualization, the distance between the drain and gate is reduced to 300 nm, while the distance between the drain and source remains at 50 nm.


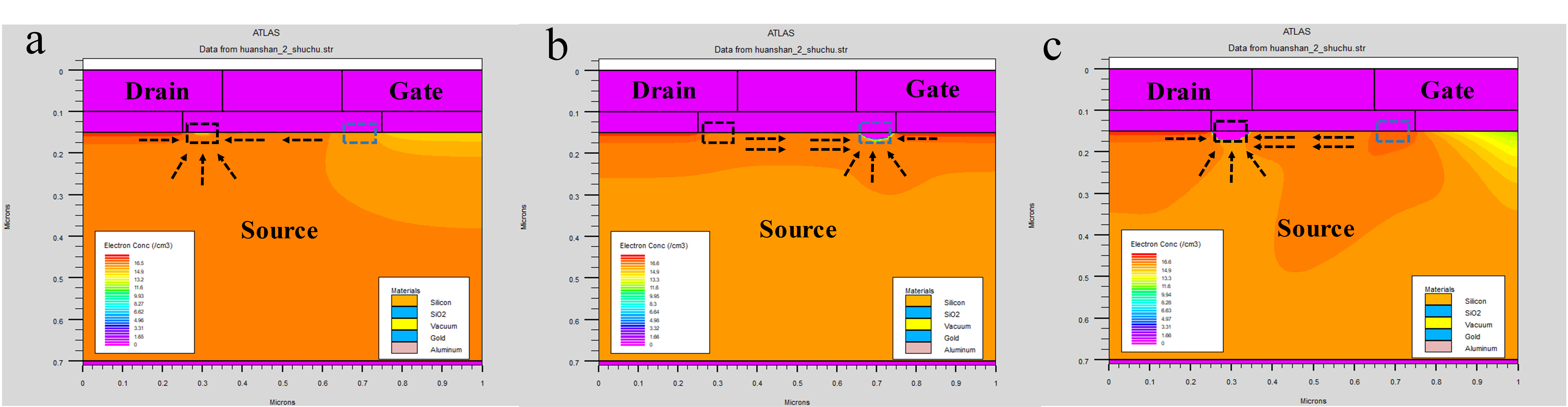


**Figure S7.** **Simulation results of the electron concentration distribution of CG-NACT for a constant drain voltage (V_d_) and different gate voltages (V_g_).** **a** Electron concentration distribution of the CG-NACT when V_d_=1.5 V and V_g_=0 V. **b** Electron concentration distribution of the CG-NACT when V_d_=1.5 V and V_g_=1.8 V. **c** Electron concentration distribution of the CG-NACT when V_d_=1.5 V and V_g_=-1.6 V. The black boxes in the figure are the regions where FN tunneling occurs at the drain and source, and the blue boxes are the regions where FN tunneling occurs at the gate and source. The black arrows represent the direction of electron movement within the semiconductor.


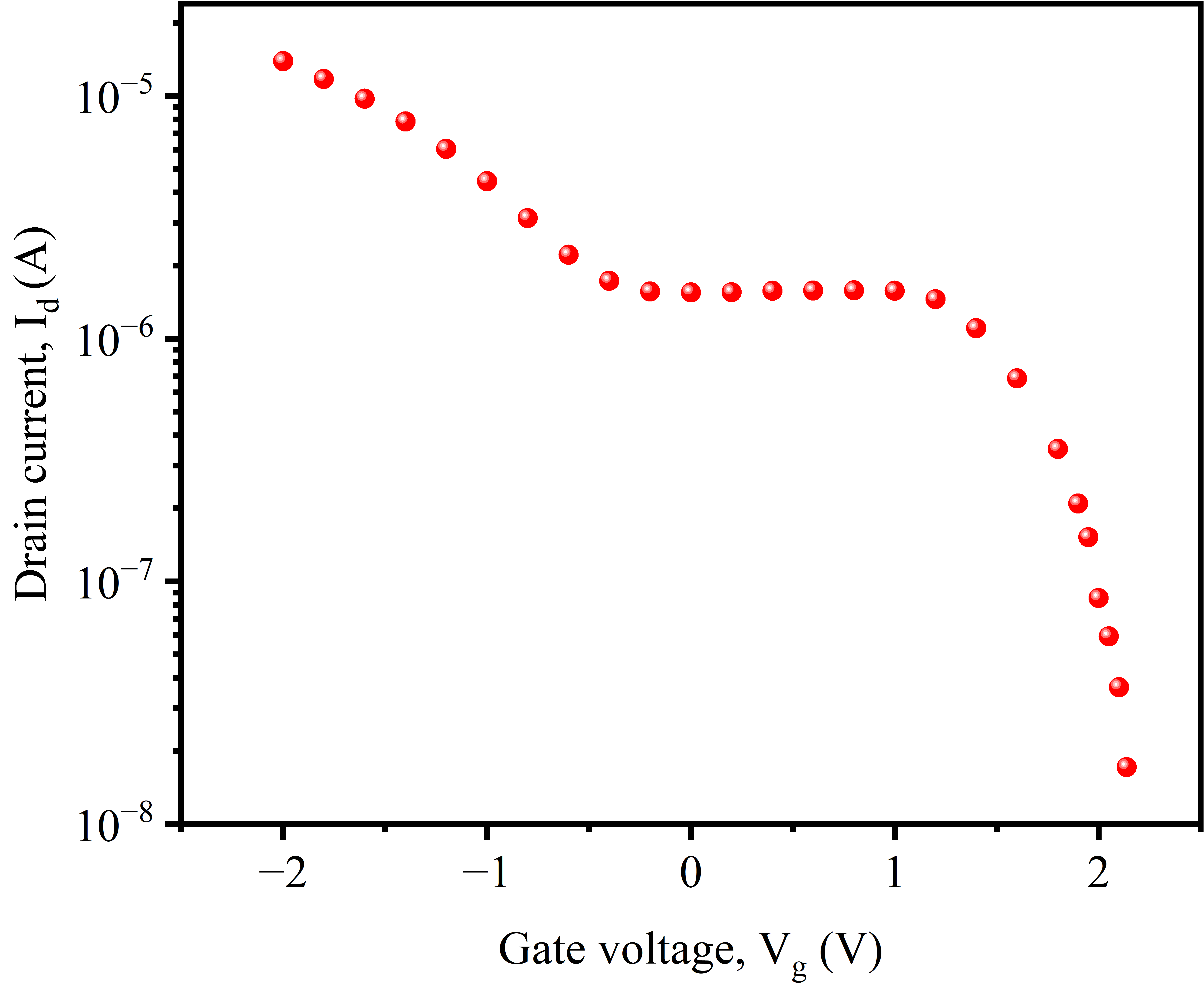


**Figure S8. Simulation of transfer characteristic of the CG-NACT with a drain voltage of V_d_=1.5V.**


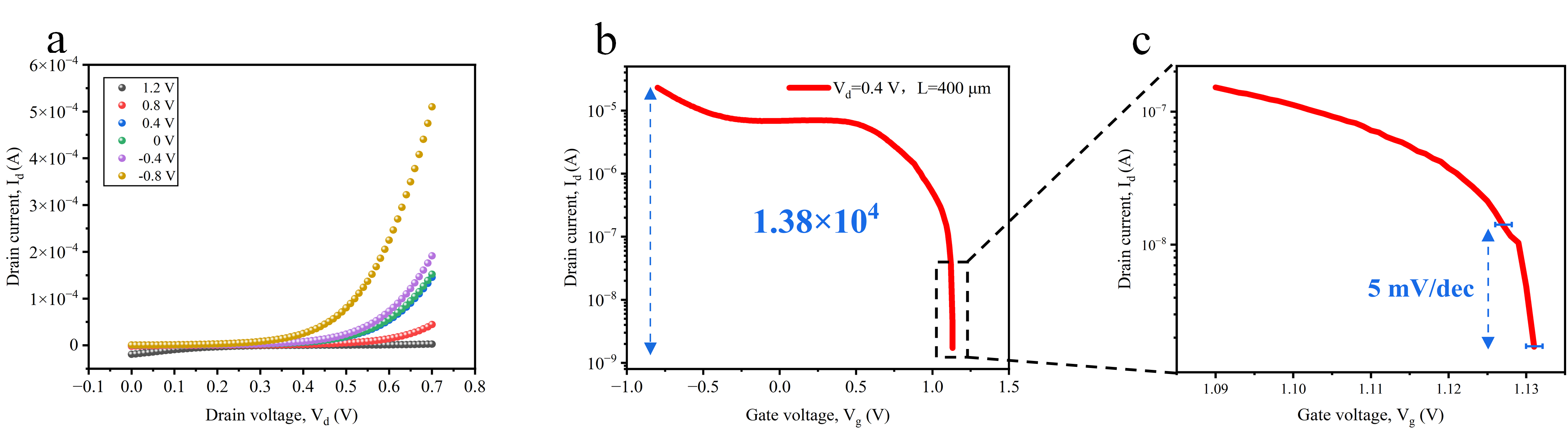


**Figure S9. Electrical properties of the D2 device (L=400 μm, BOE 10s, effective emitting areas: 240 μm^2^)**. **a** Output characteristic. **b, c** Transfer characteristic (b) and its magnified figure (c).

**
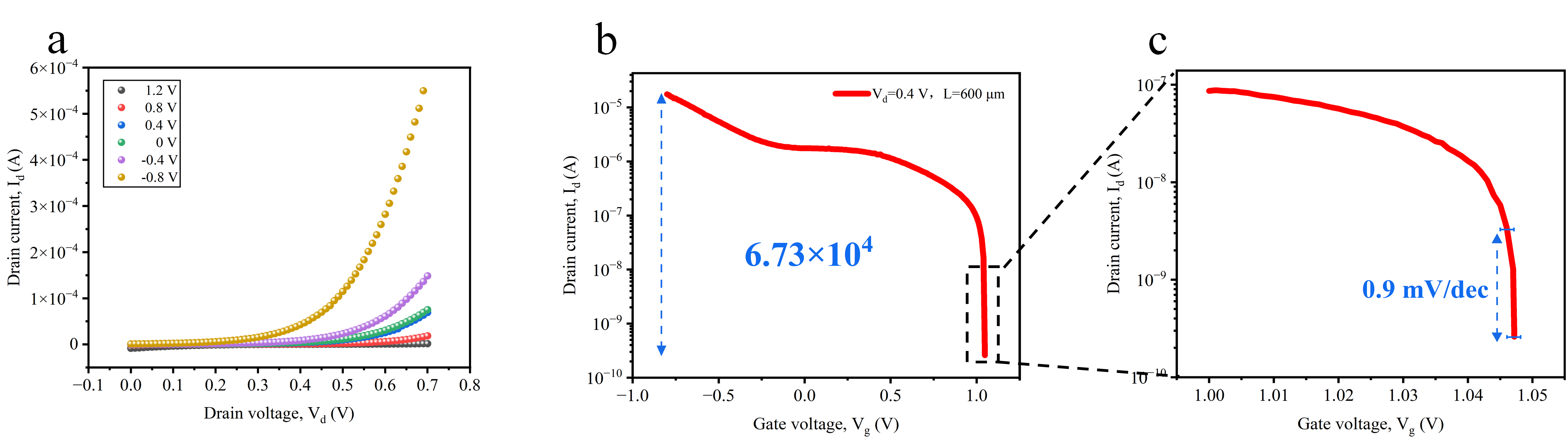
**

**Figure S10. Electrical properties of the D3 device (L=50 μm, BOE 13s, effective emitting areas: 36 μm^2^)**. **a** Output characteristic. **b, c** Transfer characteristic (b) and its magnified figure (c).


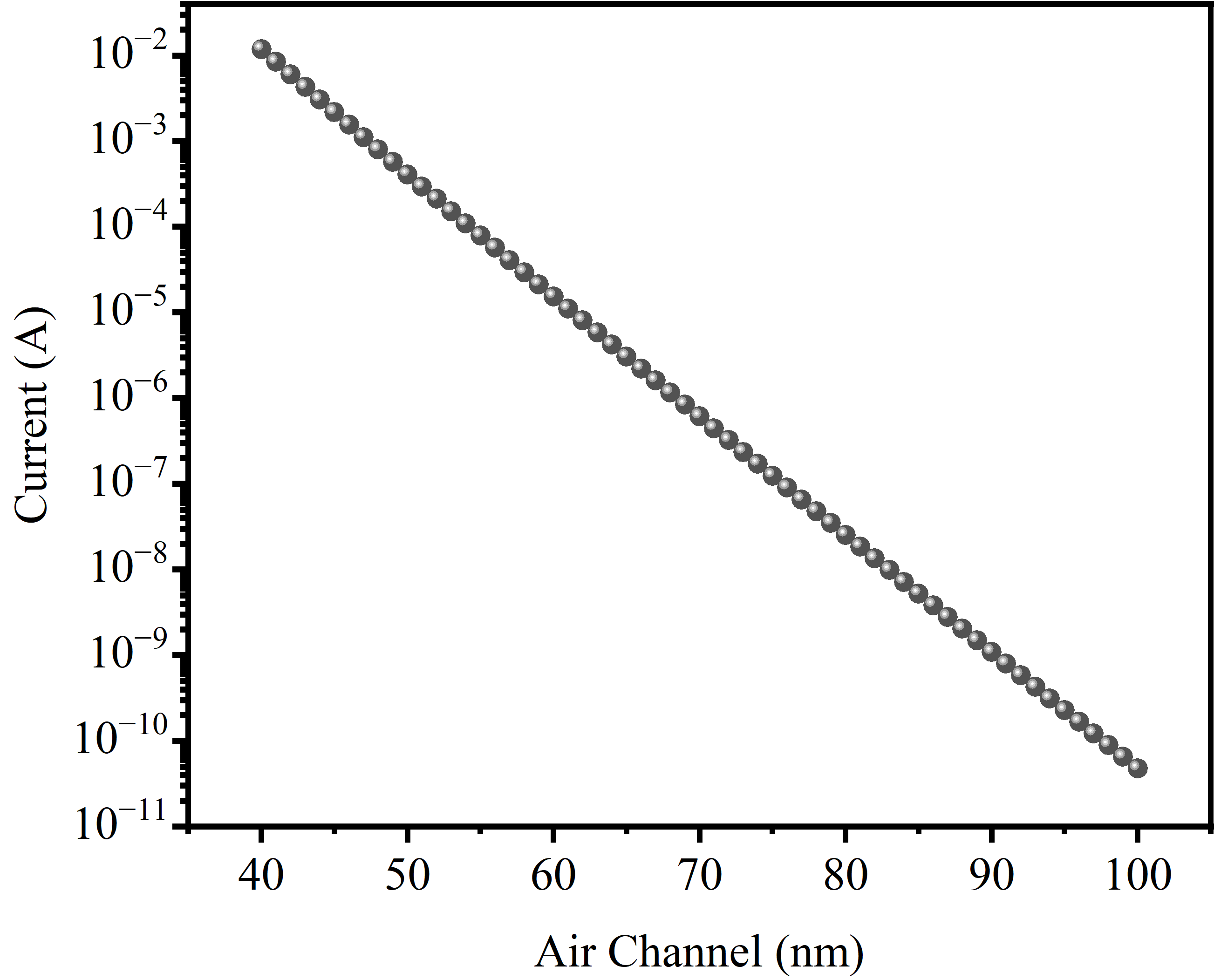


**Figure S11.** **Simulation of the effect of different air channel sizes on field emission current.**


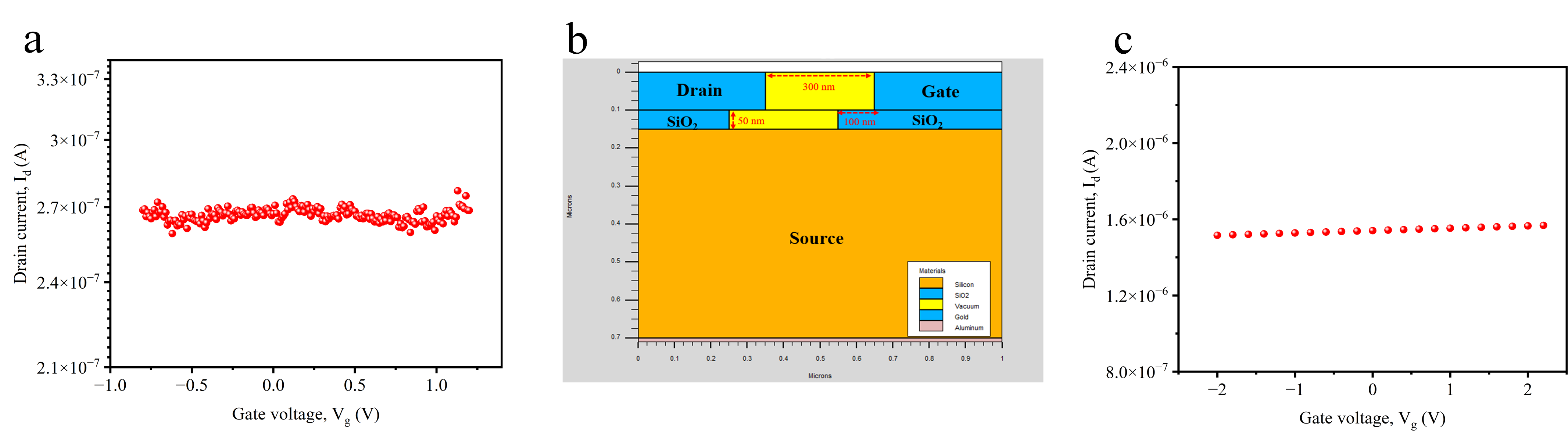


**Figure S12. Experiment and Simulation of the electrical performance of CG-NACT when there is no FN tunneling between the gate and source.** **a** Transfer characteristic of CG-NACT fabricated by the photoresist overlay process **b** Device structure of CG-NACT when there is no tunneling between the gate and source. **c** Simulation of the transfer characteristic of the CG-NACT when the drain voltage V_d_=1.5V.


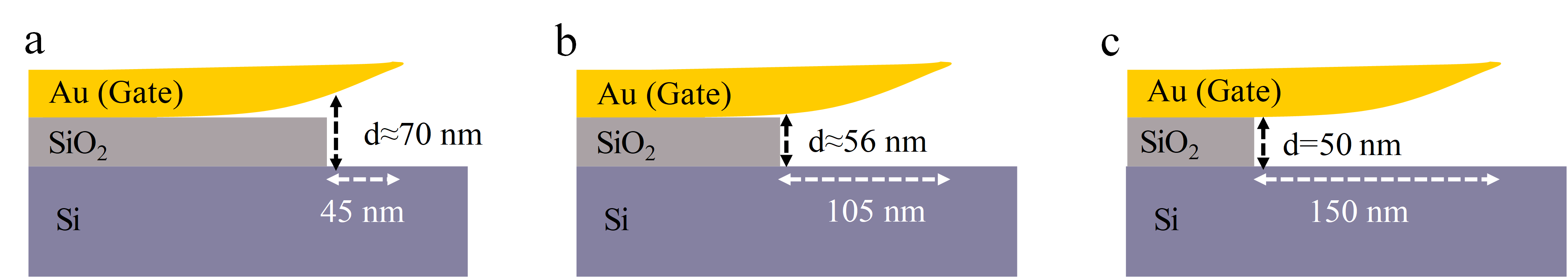


**Figure S13.** Schematic representation of nanoscale air channel formed at different etching times. **a** Wet etching for 3 s. **b** Wet etching for 7 s. **c** Wet etching for 10 s.

**
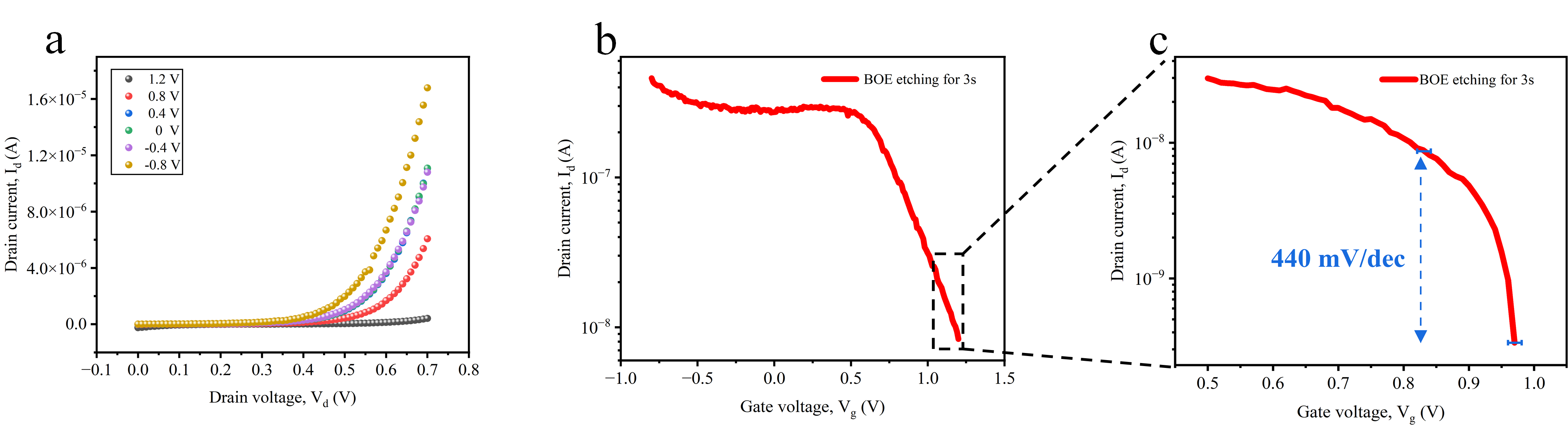
**

**Figure S14.** **Electrical properties of CG-NACT with BOE wet etching of 3s.** **a** Output characteristic. **b, c** Transfer characteristic (**b**) and its magnified figure (**c**).


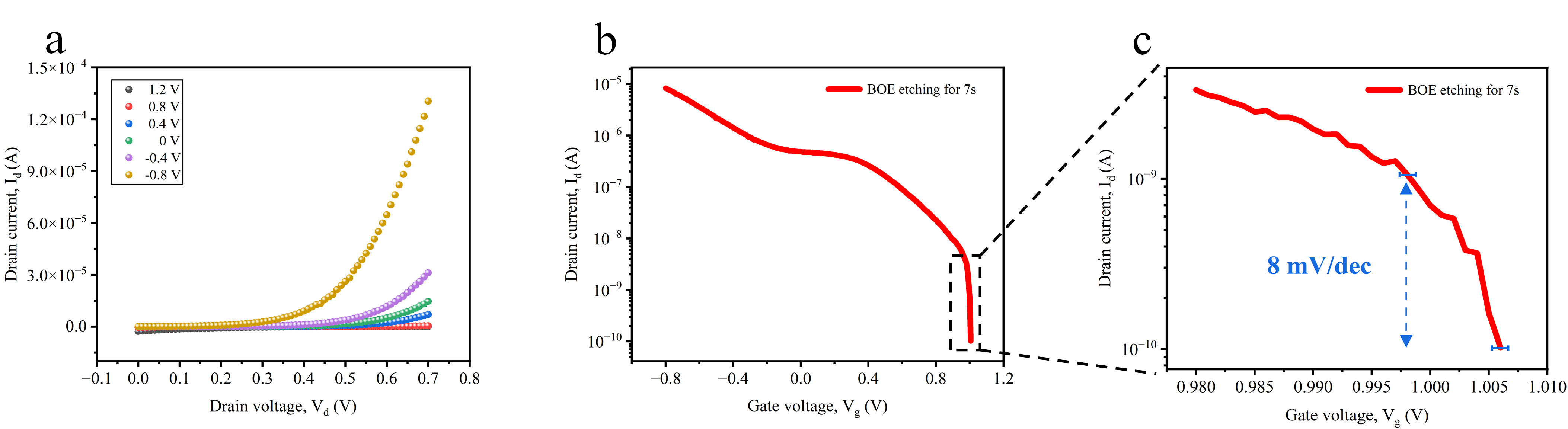


**Figure S15. Electrical properties of CG-NACT with BOE wet etching of 7s. a** Output characteristic. **b, c** Transfer characteristic (**b**) and its magnified figure (**c**).


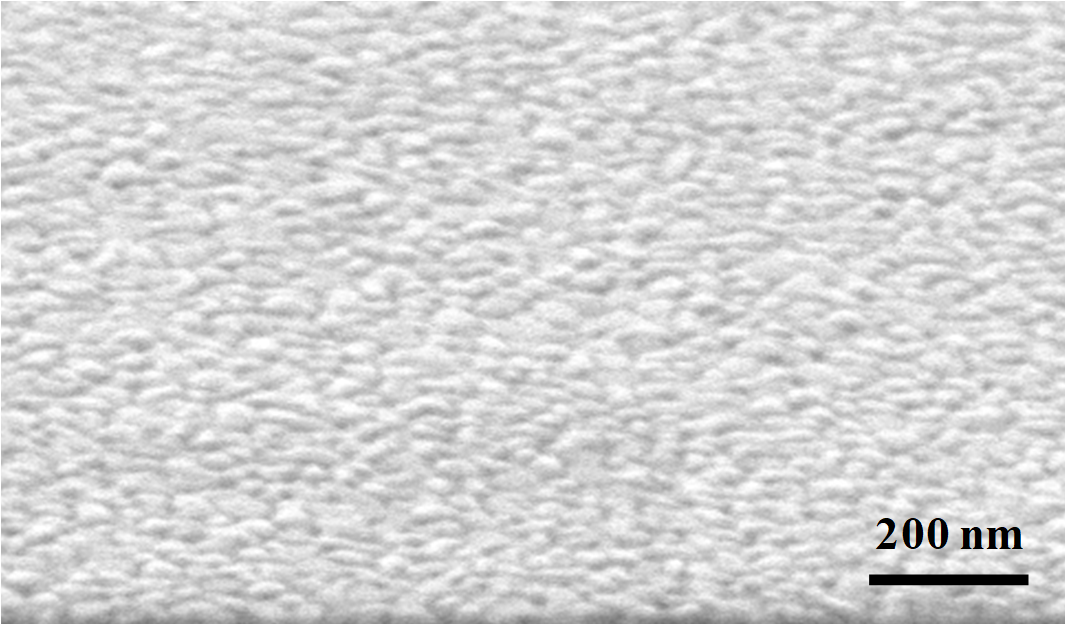


**Figure S16. Surface roughness of electron beam vaporized electrodes.**


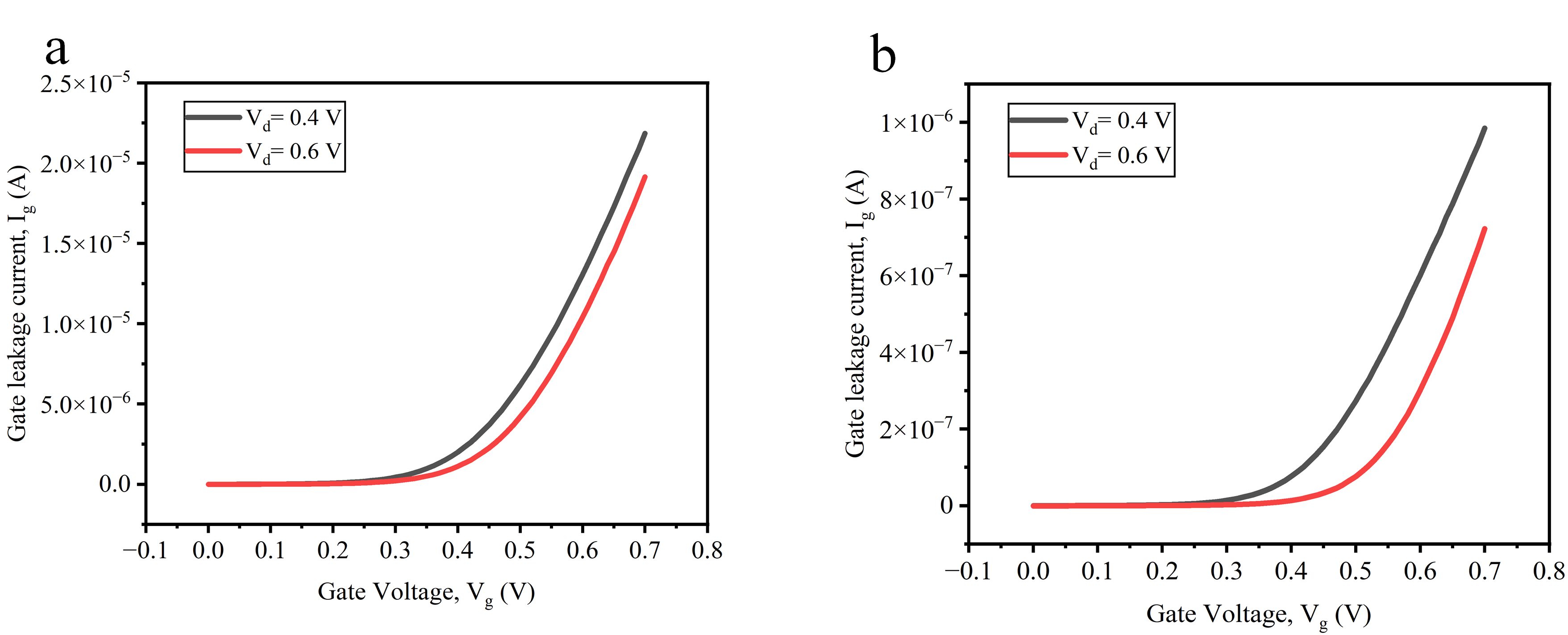


**Figure S17. Gate leakage current of D1 device (L=1000 μm, BOE 10s, effective emitting areas: 600 μm^2^) and D3 device (L=50 μm, BOE 13s, effective emitting areas: 36 μm^2^).**


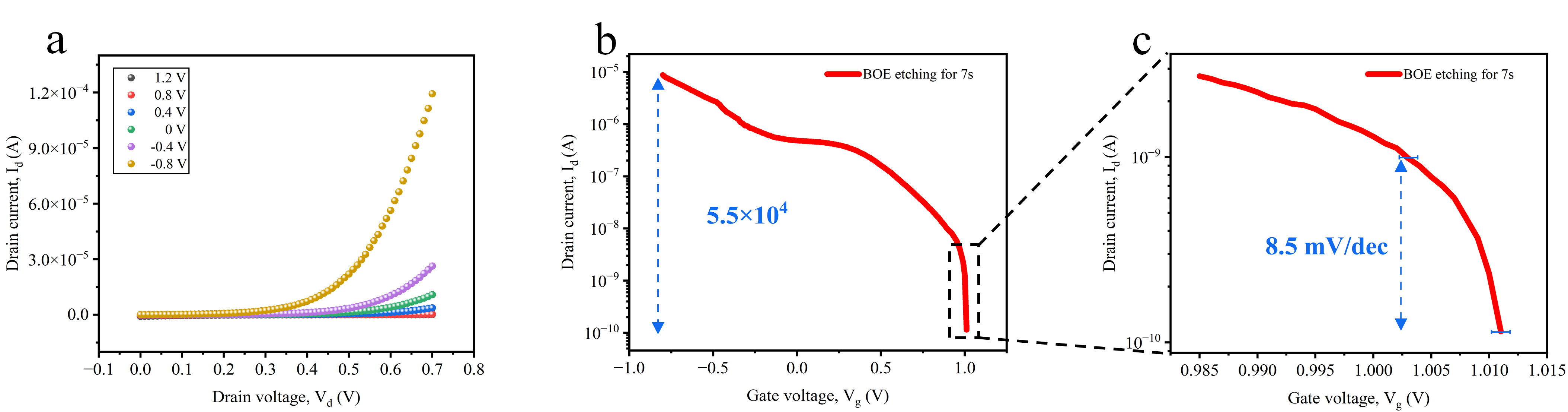


**Figure S18. Electrical properties of the D2 device (BOE 7s, effective emitting areas: 168 μm^2^) after neutron irradiation with a total dose of 1×10^10^ n/cm^2^. a** Output characteristic. **b, c** Transfer characteristic (b) and its magnified figure (c).


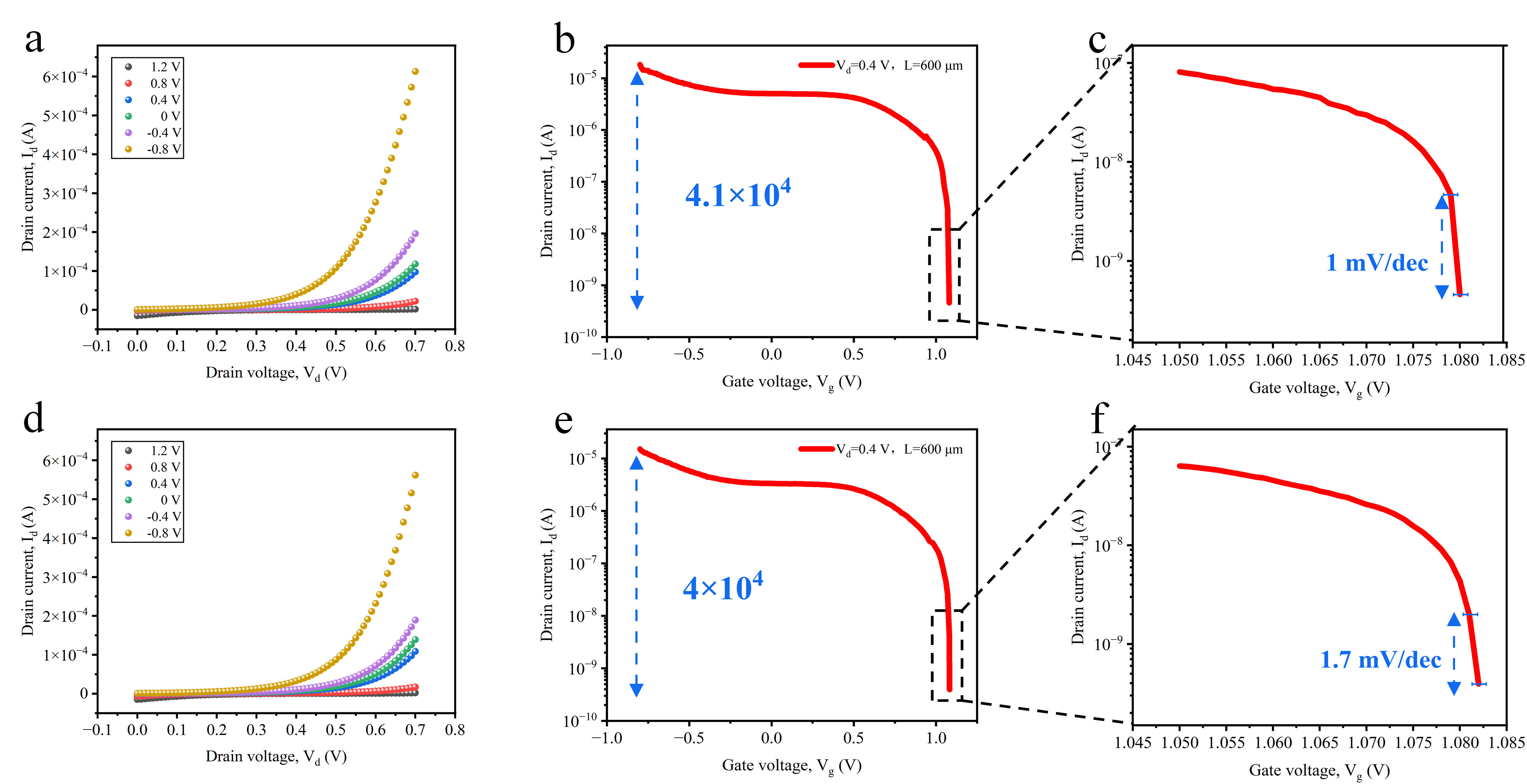


**Figure S19.** **Drain current and SS of D4 (BOE 10s, effective emitting areas: 360 μm^2^) devices before and after neutron irradiation with a total dose of 1×10^10^ n/cm2. a** Output characteristic before neutron irradiation. **b, c** Transfer characteristic (b) and its magnified figure (c) before neutron irradiation. **d** Output characteristic after neutron irradiation. **e, f** Transfer characteristic (e) and its magnified figure (f) after neutron irradiation.

**
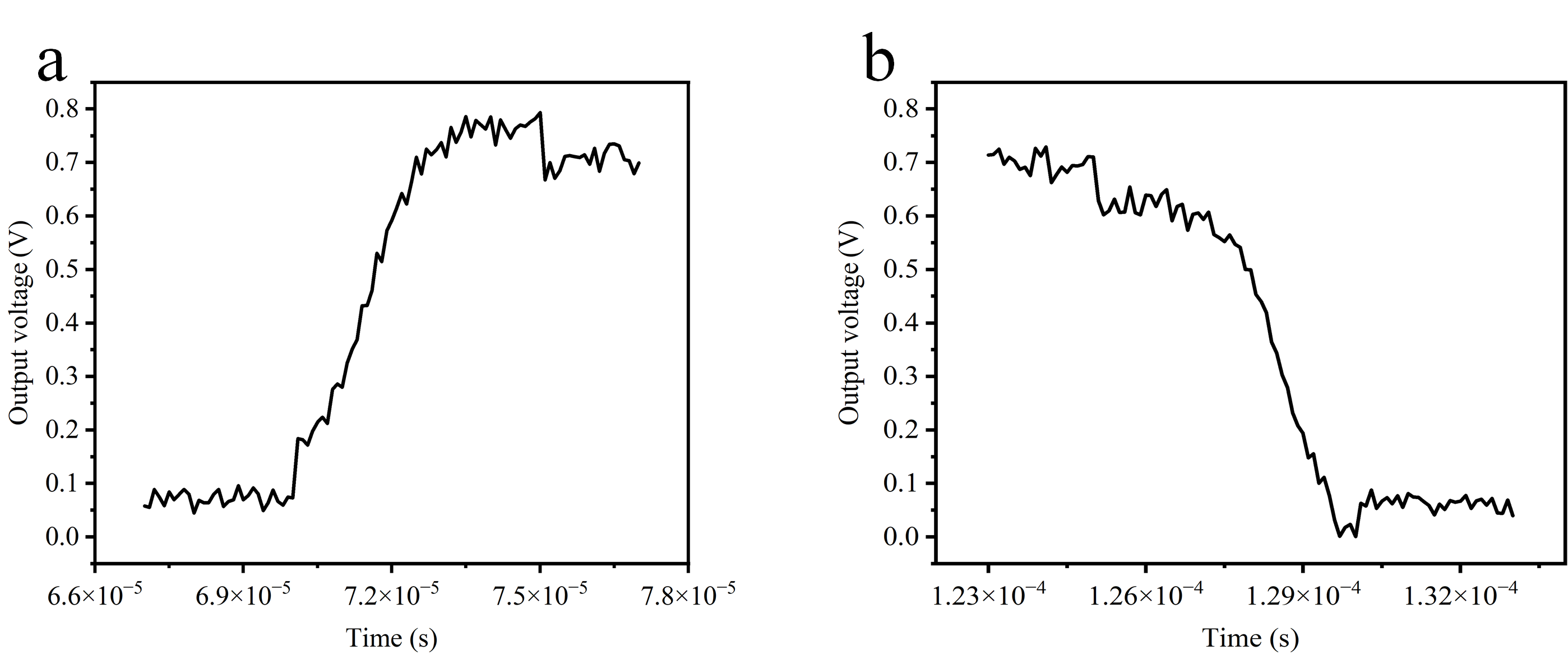
**

**Figure S20. The response time of the CG-NACT in an inverter circuit as an inverter. a** Response time of CG-NACT when the rise time of the output signal is 3 μs at a frequency of 10kHz. **b** Response time of CG-NACT at a frequency of 10kHz with a fall time of 3 μs for the output signal.


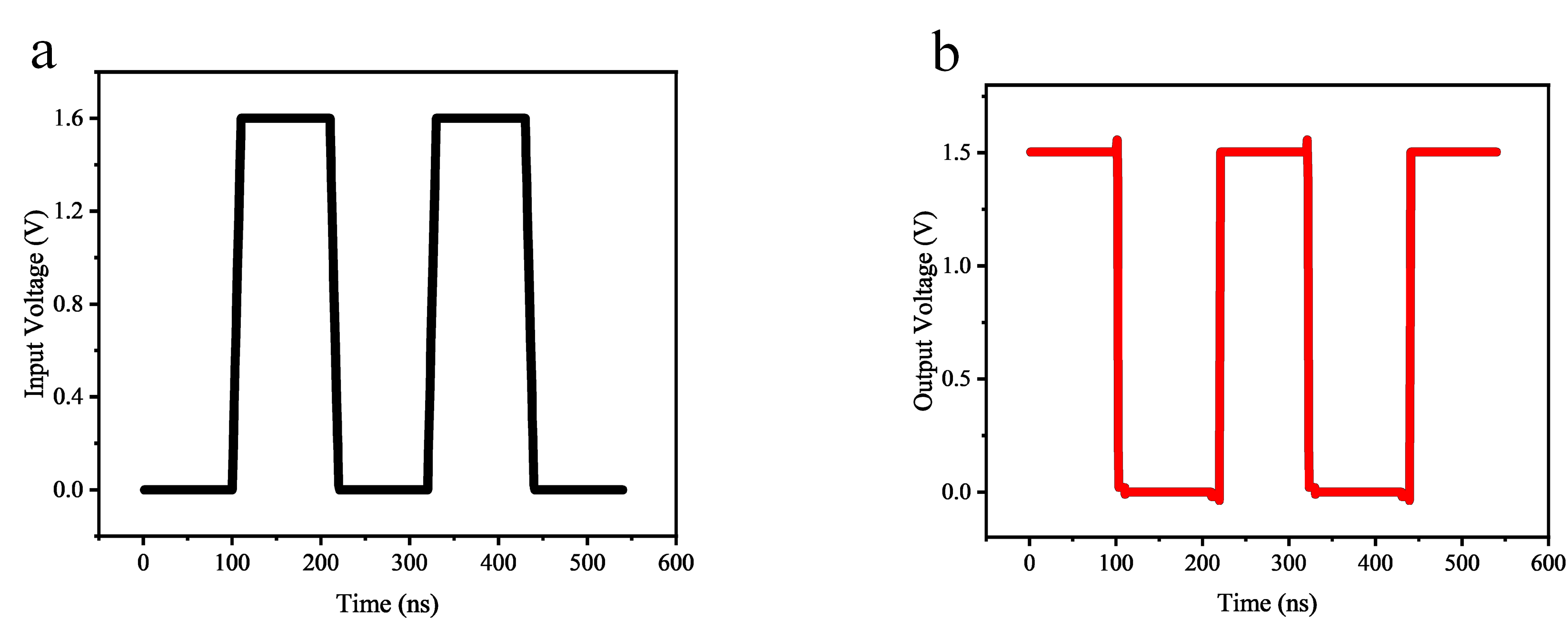


**Figure S21.** **Simulation of the electrical performance of the device as an inverter. a** Input pulse signal with a rising edge of 10 ns. **b** Output signal with a rising edge of 10 ns.

**
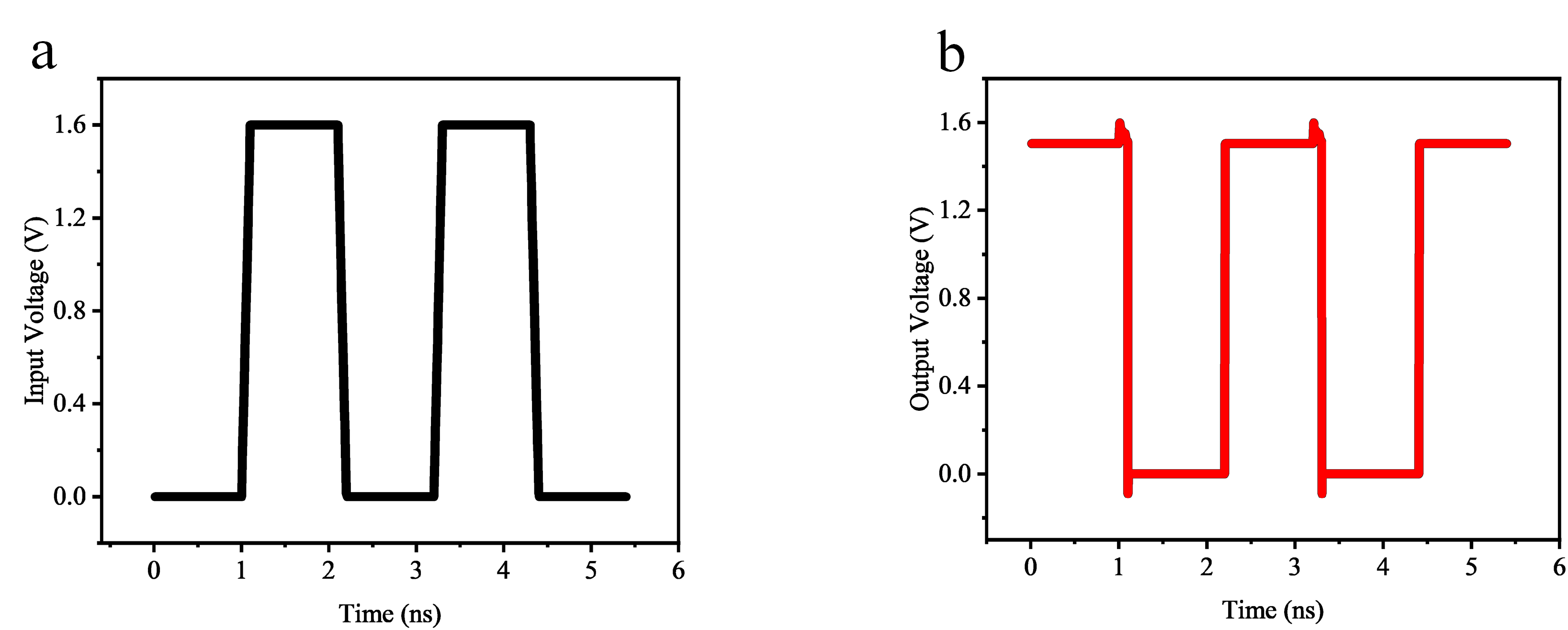
**

**Figure S22.** **Simulation of the electrical performance of the optimized device as an inverter. a** Input pulse signal with a rising edge of 100 ps. **b** Output signal with a rising edge of 100 ps.

**Supporting Notes**

1. **Schottky emission theory**

The expression for Schottky emission is given by^[7]^:

$$I=SAT^{2}exp(\varphi-\frac{\beta E^{0.5}}{kT})$$

where, *S* is emission area, *A* is the effective Richardson constant, *T* is temperature, $\varphi$ is the work function of the electrode, $\beta$ is the relative dielectric constant of the electrode, *k* is the Boltzmann’s constant, and *E* is the electric field.

From the perspective of the energy barrier, when the electric field is below 10^9^ V/m, the energy band bending is not prominent enough for electron tunneling. However, it reduces the barrier height, allowing electrons near the electrode surface to overcome the lower energy-level barrier and reach the vacuum level, as illustrated in Figure. S23.


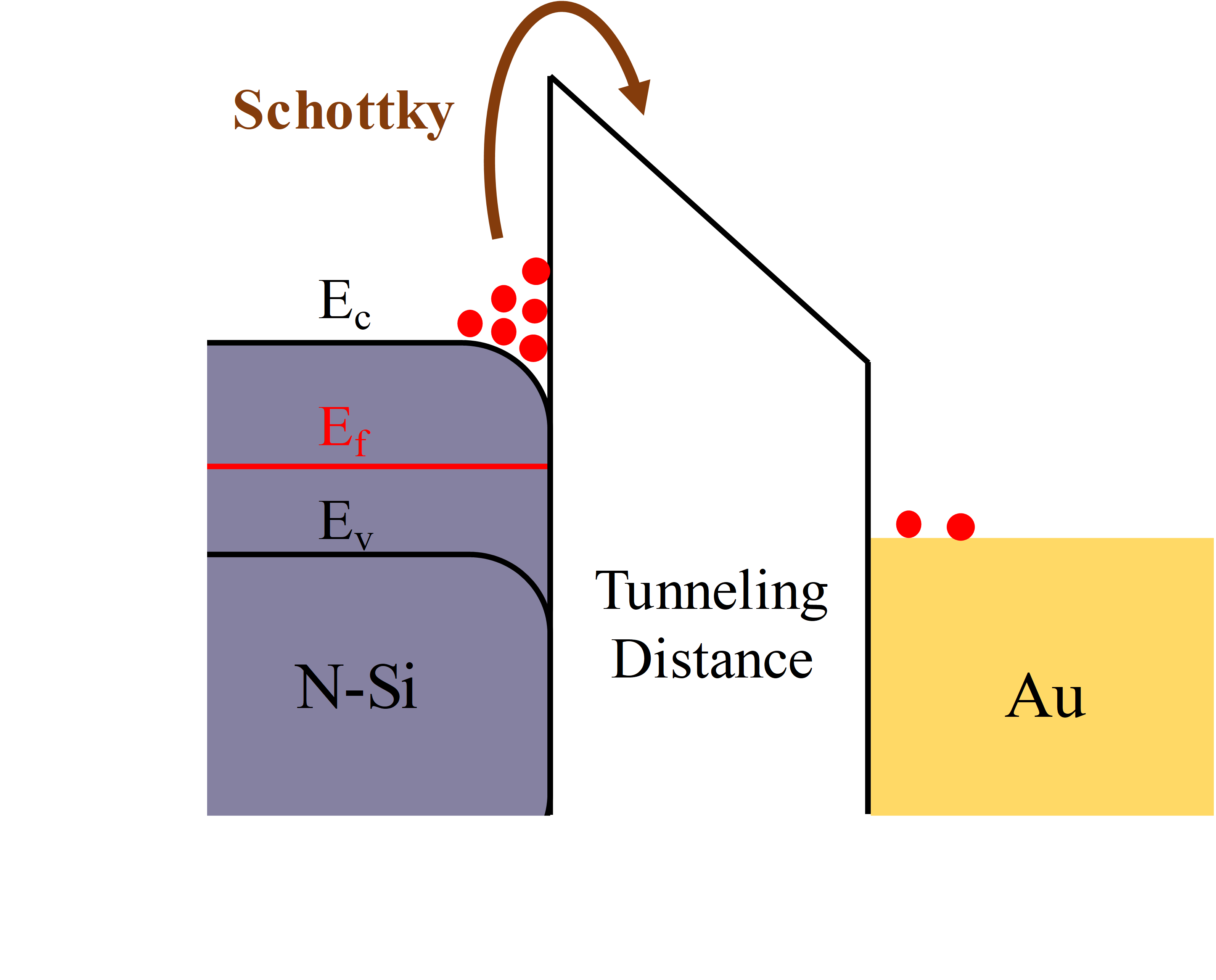


**Figure S23.** Energy band diagrams of CG-NACT with Schottky emission. E_c_, E_f_, and E_v_ denote the conduction band, Fermi level, and valence band, respectively.

1. **Fowler-Nordheim (FN) tunneling emission theory**

The expression for this phenomenon is given by^8^:

$$I=S\frac{A_{FN}\beta^{2}E^{2}}{\varphi}exp(-\frac{B_{FN}\varphi^{1.5}}{\beta E})$$

where *E* is the electric field, *S* is the area of the electrode, *φ* is the work function of the cathode material, and *β* is the field enhancement factor and *A_FN_* and *B_FN_* are the FN constants.

From the perspective of the energy barrier, when the electric field exceeds 10^9^ V/m, corresponding to the high-field regime, the energy band undergoes significant bending. This results in a triangular region where electrons can tunnel through the barrier, as illustrated in Fig. S24.


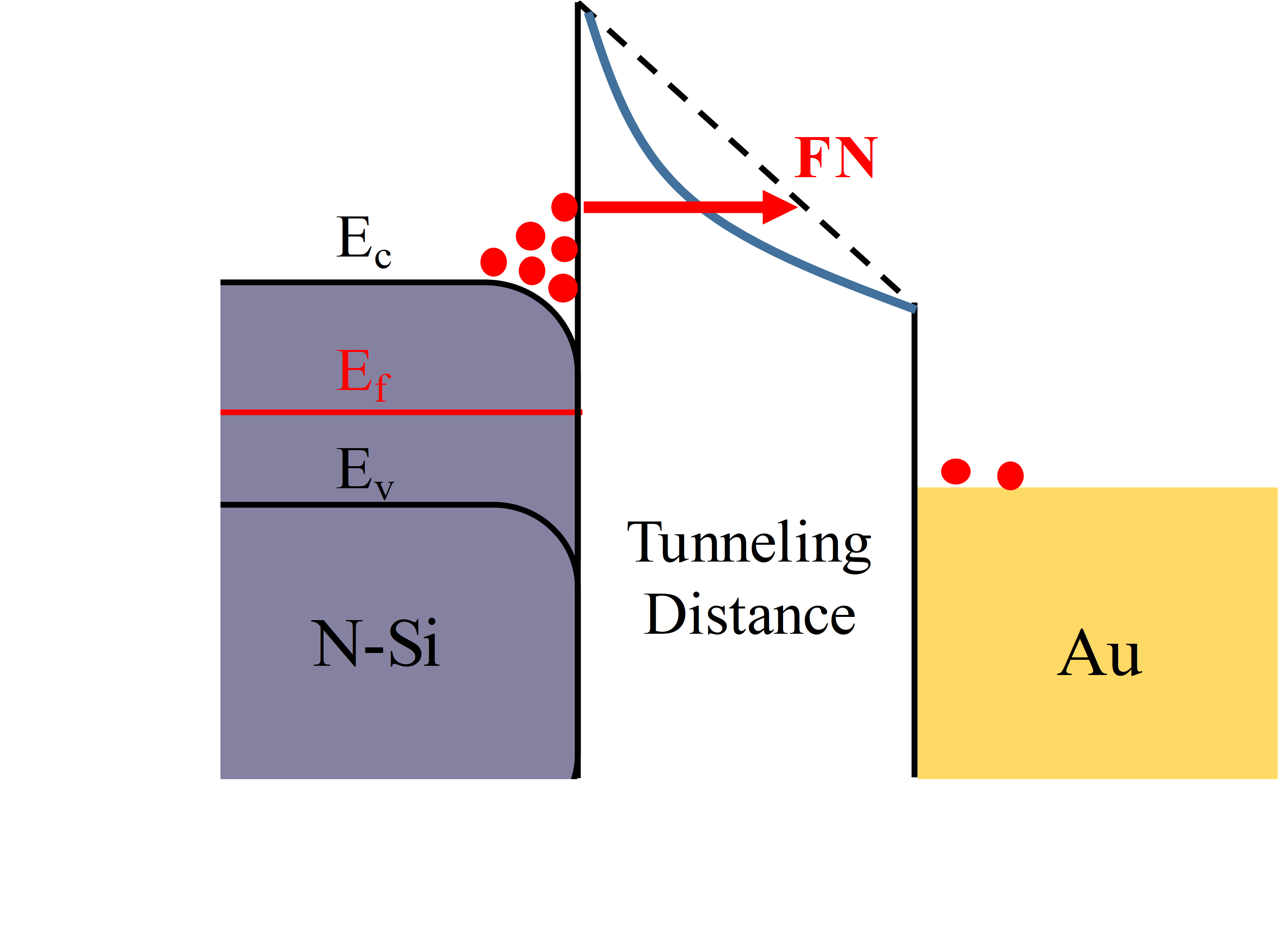


**Figure S24.** Energy band diagrams of CG-NACT with FN tunneling. E_c_, E_f_, and E_v_ denote the conduction band, Fermi level, and valence band, respectively.

**References**

1. Miao, J. et al. Heterojunction tunnel triodes based on two-dimensional metal selenide and three-dimensional silicon. *Nat. Electron.***5,** 744-751 (2022).
2. Sarkar, D. et al. A subthermionic tunnel field-effect transistor with an atomically thin channel. *Nature.***526**, 91-95 (2015).
3. Wang, X. et al. Van der Waals negative capacitance transistors. *Nat. Commun.***10,** 3037 (2019).
4. Kang, C. et al. A steep-switching impact ionization-based threshold switching field-effect transistor." *Nanoscale.* **15**, 5771-5777 (2023).
5. Wang, Y. et al. Record‐Low Subthreshold‐Swing Negative‐Capacitance 2D Field‐Effect Transistors. *Adv. Mater.* **32**, 2005353 (2020).
6. Gao, A. et al. Observation of ballistic avalanche phenomena in nanoscale vertical InSe/BP heterostructures. *Nat. Nanotechnol.* **14,** 217-222 (2019).
7. Sun, Y. H., David, A. J. & John, T. W. Y. Self-heating Schottky emission from a ballasted Carbon nanotube array. *Carbon.***58,** 87-91(2013).
8. Han, J.W., Jae, S. O. & Meyyappan, M. Cofabrication of vacuum field emission transistor (VFET) and MOSFET. *IEEE Trans Nanotechnol.* **13,** 464-468 (2014).
